# Anatomical-electrical coupling of cardiac axes: Definitions and population variability for advancing personalised ECG interpretation

**DOI:** 10.1371/journal.pcbi.1013161

**Published:** 2025-07-10

**Authors:** Mohammad Kayyali, Ana Mincholé, Shuang Qian, Alistair Young, Devran Ugurlu, Elliot Fairweather, Steven Niederer, John Whitaker, Martin Bishop, Pablo Lamata

**Affiliations:** 1 School of Biomedical Engineering and Imaging Sciences, King’s College London, London, United Kingdom; 2 BSICoS, I3A, IIS Aragon, University of Zaragoza, Zaragoza, Spain; 3 National Heart and Lung Institute, Imperial College London, London, United Kingdom; 4 The Alan Turing Institute, London, United Kingdom; Ocean University of China, CHINA

## Abstract

Electrocardiogram (ECG) recordings are affected by the heart’s three-dimensional orientation within the thorax, i.e., the anatomical axis. Various cardiac conditions can cause the anatomical axis to shift and/or alter the pattern of electrical activation, leading to changes in the electrical axis. Nevertheless, there remains a lack of a formal, population-level study of the interplay between the cardiac anatomical and electrical axes and the factors that affect them. In this context, this study aimed to: (1) propose standardised definitions for the cardiac anatomical and electrical axes, (2) characterise their population-wide interplay in healthy conditions, (3) evaluate the impact of hypertension on their distribution and (4) identify associations with phenotypical and disease characteristics. Using cardiac magnetic resonance images and 12-lead ECGs from ~39,000 UK Biobank participants, patient-specific, paired biventricular geometries and vectorcardiograms were constructed. Five anatomical and four electrical axis definitions were computed, with the optimal pair of definitions selected based on their mutual alignment in 3D space within 28,000 healthy subjects. Accordingly, the anatomical axis was defined as the vector from the apex to the spatial centre of the four valves, and the electrical axis as the direction of the maximum QRS dipole. Mean angular separation in 3D, $\Delta AE_{3D}$, was 145.0° ± 16.8° in the healthy cohort. The electrical axes exhibited a much larger variability, and strong evidence of anatomical-electrical coupling was identified. Increasing BMI notably affected the anatomical axis, rotating the heart more horizontally—a pattern mirrored by the electrical axis. Both axes were also significantly influenced by sex and, to a lesser extent, age. The axes were then studied in the sub-cohort of ~3,500 UK BioBank participants with primary hypertension, where a similar rotational pattern as that with increasing BMI was revealed. Finally, phenome-wide association studies in the 39,000 participants reveal associations between the axes angular metrics and phenotypes signalling an increased afterload, and an association to hypertension among other clinical conditions. These findings underscore the complex anatomical-electrical interplay and highlight the potential of cardiac axes biomarkers for an improved clinical ECG interpretation and disease characterisation.

## Author summary

Electrocardiograms (ECGs) are among the most widely used tools in cardiac diagnostics, capturing the electrical potential on the skin’s surface that reflects the heart’s electrical activity. This signal is influenced by the heart’s orientation and position within the chest—factors often overlooked during ECG interpretation. In this study, we examined the anatomical axis as the heart’s orientation and the electrical axis as a 3D spatial representation of the heart’s electrical activity, exploring their interactions in both healthy individuals and those with hypertension. Using cardiac magnetic resonance imaging and ECGs from about 39,000 UK Biobank participants, we investigated how factors such as body mass index, age, sex, and hypertension affect these axes and their coupling. We found that increased BMI, male sex, and hypertension are associated with a more horizontal heart orientation and electrical axis, emphasising complex anatomical-electrical interaction. Our findings also show association with health outcomes, pointing out the importance of accounting for patient-specific variations and suggest that considering these factors could lead to more accurate and personalised ECG interpretation.

## 1. Introduction

The electrocardiogram (ECG) is a cornerstone of cardiac diagnostics, and its ubiquity in clinical practice emphasises the importance of accurate ECG interpretation. The ECG captures the body surface electrical field, which is a dynamic representation of cardiac electrical activity and is considerably shaped by the heart’s anatomical characteristics. The heart’s size, position, and orientation within the thorax significantly influence the propagation and detection of these electrical signals, shaping the resultant ECG waveforms. Thus, these waveforms we observe are not just a reflection of the heart’s electrical state, but also a product of its geometrical attributes and spatial relationship with surrounding structures.

A primary challenge in ECG interpretation lies in distinguishing between ECG variations due to anatomical variability and those indicative of pathology. The emergence of cardiac digital twins – patient-specific computational models that integrate cardiac anatomical, physiological and temporal information – offers opportunities to address this challenge by creating digital representations of both static and dynamic features of the heart, thereby facilitating investigations of how these aspects interact and influence clinical measurements [1]. These in-silico approaches offer a deeper understanding of the anatomical-electrical relationship by exploring the impact of cardiac anatomy on measured ECG parameters such as QRS duration and wave amplitudes [2,3]. However, there remains a notable lack of population-wide information on this relationship, hindering the translation into personalised, practical applications.

Central to understanding the anatomical-electrical interplay are the concepts of anatomical and electrical axes. Traditionally, the anatomical axis has been manually derived from imaging data as the heart’s long axis [4], often overlooking the right ventricle’s contribution. The electrical axis, typically derived using Einthoven’s triangle [5], is limited by its reliance on frontal plane projections. Nevertheless, automated reconstruction of cardiac anatomy from cardiac magnetic resonance images (cMRIs), combined with comprehensive electrical representations like the vectorcardiogram (VCG) [6], enables large-scale and patient-specific studies that integrate personalised anatomical data and static representations of the cardiac electrophysiological data.

Despite several studies examining cardiac anatomical and electrical axes [7–9], no standardised axes definitions exist, nor is there a population-wide quantification of anatomical-electrical interplay and its coupled or individual variability. This study investigates the anatomical-electrical relationship across a population of about 39,000 participants from the UK Biobank, using participant cMRIs and ECGs. We seek to propose standard definitions of cardiac axes that maximise anatomical-electrical axes alignment in 3D. Additionally, we characterise the interplay of the axes and their interaction with demographic variables under healthy conditions and provide insight into the effect of primary hypertension. Finally, the associations of axes angular metrics with phenotypes and clinical diagnoses are explored in a phenome-wide association study. This comprehensive analysis of anatomical-electrical coupling aims to provide a foundation for patient-specific clinical care where individual cardiac orientation can inform personalised interpretation of cardiac electrical activity.

## 2. Methods

### 2.1. Clinical data

The source of clinical data in this study is the UK Biobank, which is a population-based database and research resource, providing biomedical information for around half a million adult volunteers in the UK. Volunteers were recruited in 2006–2010 and gave consent for the collection and storage of their biomedical data for future research analysis. The data includes heart and brain imaging, lifestyle questionnaires, physical measures, and cognitive and genetic information. Further details on the study design and conduct have been described [10,11]. The study exclusively used data previously collected by UK Biobank, with no additional live participants.

### 2.2. cMRIs and 3D reconstruction

Standardised protocol was applied by UK Biobank for acquiring cMRIs, with ECG gating, for all participants. The comprehensive description of the cMRI protocol, including scanner properties and acquisition parameters, is described in [12].

An automated workflow facilitated the large-scale generation of ~49,000 surface meshes from cMRIs at the end diastolic phase. Details of this established workflow can be found in [13,14]. The biventricular anatomy was first segmented using nnU-net architecture [15], and an atlas-based pipeline was then employed to generate the 3D surface meshes from the labelled anatomical regions [16], including each of the four valves and the left and right ventricles. Subsequently, an automatic quality control check was employed to avoid the inclusion of abnormal flat meshes: the sphericity index (SI), defined as the ratio of short to long axis, was a robust metric to detect these instances. Any mesh with an SI ± 3 SD from the population mean was deemed abnormally flattened and excluded, accounting for <0.5% of total meshes. Manual review confirmed these distortions, justifying their removal. Examples and further details of the excluded meshes are provided in S1.1. and Fig A in S1 Appendix.

### 2.3. ECG preprocessing

Digital 12-lead (at-rest) ECGs were stored in XML files using the *GE, CASE/Cardiosoft v6* system. The setup of the limb and chest leads can be seen in [17], as used by trained members of the UK Biobank staff. For each participant, 10-second samples were acquired with a sampling frequency of 500 Hz and a resolution of 1 µV. During data acquisition, a series of filters were applied: a low-pass filter at 100 Hz, a high-pass filter at 0.1 Hz, and a 50 Hz power-line noise filter. No additional preprocessing was applied in this study.

The median beat was extracted and solely used in this study’s analysis. To exclude low quality ECGs, a standard method based on QRS amplitude was used. The population mean of QRS amplitude (maximum deflection from isoelectric line) was calculated for each lead. ECGs were omitted if the QRS amplitude exceeded ±3 SD from the population mean in any lead. This QRS amplitude-based exclusion was selected as it reliably identifies significant deviations attributable to noise, lead misplacement, or recording errors [18,19]. This approach was also used by a prior large-scale, UK Biobank ECG analysis study [20].

### 2.4. Study population

**Fig 1:**
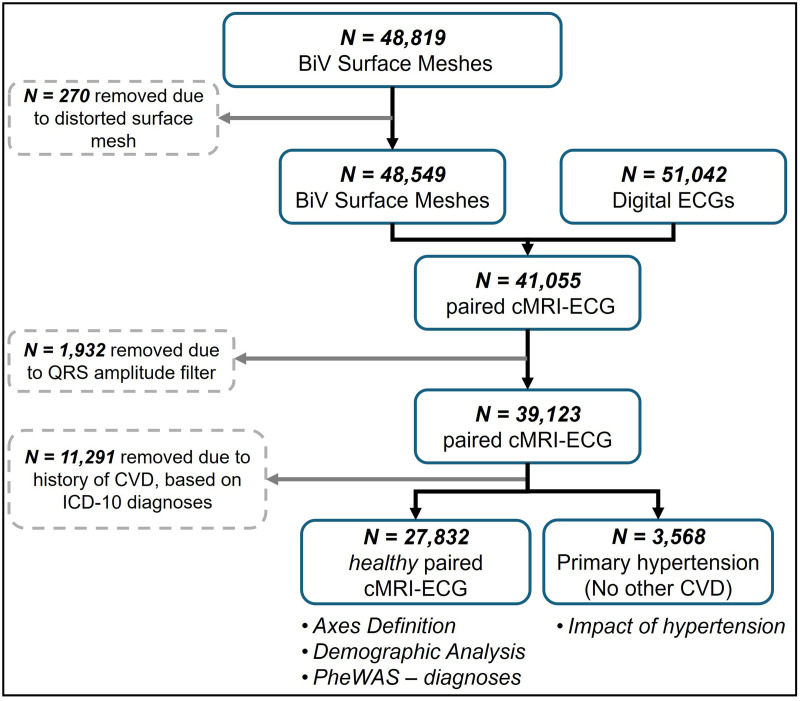
Flow chart for the inclusion of UK Biobank subjects with paired cMRI and ECG with the respective studies for each cohort.

A flow chart of the participant inclusion is shown in Fig 1. From 41,055 subjects with paired cMRI and ECG, 1,932 were excluded based on ECG quality filtering. Healthy patients were identified using the UK Biobank diagnoses data, provided as codes from the International Classification of Diseases – 10^th^ edition (ICD-10). Subjects with diagnoses of ‘Diseases of the Circulatory system’ (I00-I99) were excluded. A detailed description of how healthy patients were filtered can be found in S1.2 in S1 Appendix.

### 2.5. Cardiac axes definitions

For a comprehensive analysis, multiple definitions were used to compute anatomical and electrical axes. Table 1 outlines the definitions of these axes, which were derived from the surface meshes for anatomical axes and from the vectorcardiograms for electrical axes.

**Table 1:** Summary of the different definitions of cardiac axes investigated.

Anatomical Axis		
1	${PC1}_{LV}$	Principal axis of LV endocardial surface
2	${PC1}_{LRV}$	Principal axis of biventricular epicardial surface
3	MVA	Axis connecting apex to centre of mitral valve
4	MAVA	Axis connecting apex to centre of mitral and aortic valve
5	VPA	Axis connecting apex to the centre of all four valves
Electrical Axis		
1	maxQRS	Dipole with the maximum magnitude
2	meanQRS	Mean dipole across QRS loop
3	v−avgQRS	velocity-weighted mean dipole vector across the QRS loop
4	eig1QRS	eigenvector from largest eigenvalue of QRS loop SVD

#### 2.5.1. Anatomical axes computation.

**Fig 2:**
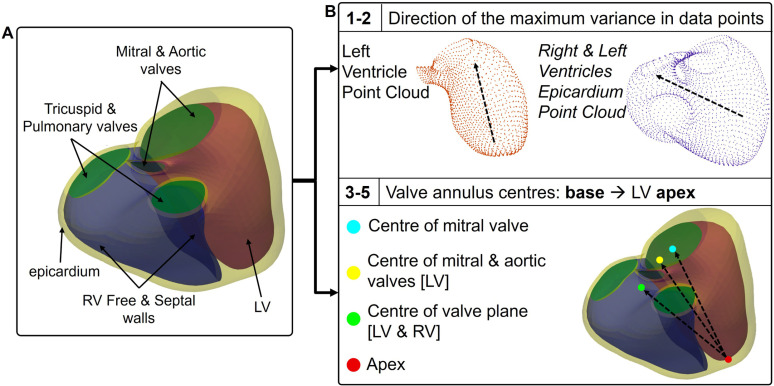
Anatomical axes computation methods. **(A)** The labelled anatomical regions of the surface meshes as outputted from the automated pipeline. **(B)** The 5 distinct definitions used to compute the axes, based on PCA’s first component of biventricular point clouds and different combinations of labelled valvular regions. Note that the apex will have a slightly different location for each of the 3 axes definitions, but it has been drawn as a single one for simplicity of the figure.

Surface mesh analysis was performed in Python using PyVista [21]. Five orientation axes were computed for each biventricular anatomy based on labelled anatomical regions and principal component analysis (PCA). These definitions are visualised in Fig 2. Two axes of inertia were derived from applying PCA to the point cloud of the surface mesh exclusively. The principal (first) component was used to calculate both axes: ${PC1}_{LV}$ from the point cloud of the left ventricle’s endocardial surface, and ${PC1}_{LRV}$ was computed from the point cloud of the epicardium, encompassing both the left and right ventricles. Three additional axes were derived from apico-basal pairs of reference landmarks: three definitions for the basal landmark were established, corresponding to the midpoint (i) of the mitral valve, (ii) of the mitral and aortic valves, or (iii) of all four valves (i.e., the centre of the valve plane). The apex was then defined as the furthest point, on the left ventricular endocardial surface, from each basal landmark. Finally, the anatomical axis was defined as the vector from the apical to the basal landmark with the following configurations: (i) Centre of mitral valve - apex, *MVA* (ii) Centre of mitral and aortic valves - apex, *MAVA* (iii) Centre of valvular plane - apex*, VPA.*

#### 2.5.2. Electrical axes computation.

The Kors transformation was used to construct the vectorcardiogram (VCG) from the median ECG, with three orthogonal leads: $V_{x}$, $V_{y}$, and $V_{z}$ [22]. The VCG lead vectors correspond to those of Frank’s lead system, which align with the body’s three main axes and are normalised in lead strength [23]. The dynamic x, y, and z components form the heart vector, which represents the resultant cardiac dipole at each timepoint and indicates changes in magnitude and direction throughout the cardiac cycle. These factors are fundamentally not represented in the scalar ECG, where only the magnitude along a certain lead can be known. The VCG thus caters to a 3D representation of the heart’s underlying electrical activity.

**Fig 3:**
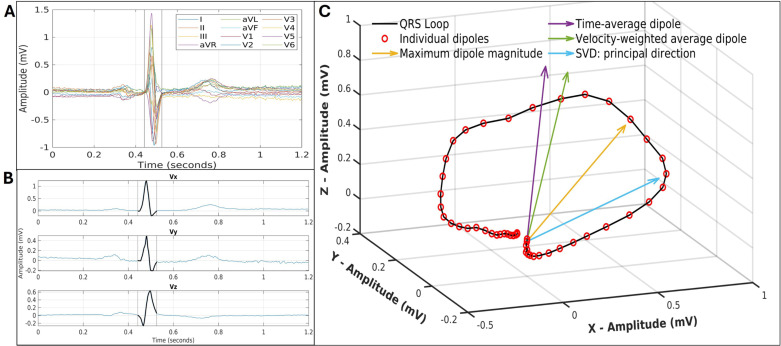
Construction of vectorcardiogram for electrical axis definitions. **(A)** Median Beat of 12-lead ECG. **(B)** Orthogonal leads of VCG with the QRS segment in black. **(C)** 3D QRS loop with 4 definitions of the electrical orientation axis.

This study focused on the QRS complex, due its diverse morphology variation in relation to pathology and heart geometry. The QRS loop from the VCG was isolated using the automatically computed QRS onset and offset times (from the acquisition software). QRS onset corresponded to the 3D origin point (0,0,0). From this loop, four electric orientation axes were computed using the dipole orientation and magnitude variations of ventricular depolarisation, as shown in Fig 3.

The dipole magnitude, ${\left({V_{x}}^{2}+{V_{y}}^{2}+{V_{z}}^{2}\right)}^{1/2}$, was calculated for each time point, and maxQRS was identified as the dipole with the maximum magnitude. The mean x, y, z components across the QRS complex were used to construct the vector meanQRS. These two methods represent the electrical wavefront’s average direction during depolarisation [6,24]. The velocity of the cardiac dipole movement varies across the QRS segment in a regular manner [25]. Hence, there is a larger concentration of vectors at the beginning and end of QRS loop (Fig 3C) for a constant sampling frequency. A velocity-weighted average dipole v−avgQRS, was constructed to account for the changing dipole velocity along the loop. Lastly, a method for approximating the main propagation direction was employed [26]. The x, y, z data points were centred around the mean and singular value decomposition (SVD) was applied. The primary direction, eig1QRS, was identified as the eigenvector corresponding to the largest eigenvalue of the correlation matrix. For the directional analysis, the unit vector of each dipole was used as the electrical axis direction.

### 2.6. Axes analysis

To truly map the cardiac dipole within the 3D anatomy, the VCG coordinate system was aligned with the DICOM LPS (+: Left-Posterior-Superior) coordinate system of the cMRI slices. This alignment preserved the orientation of biventricular surface meshes relative to the torso. The resulting coordinate system has the x-axis extending from right to left, the y-axis from anterior to posterior, and the z-axis from inferior to superior. Further details about the conventions used for the axes and a visualisation of their alignment are in S1.3 and Fig B in S1 Appendix.

For analysing the anatomical-electrical relationship using the cardiac axes, each vector was represented using spherical coordinates (θ, ϕ), where θ corresponds to the frontal plane angle, and ϕ represents the angle of the vector from the anterior-posterior axis (the transverse plane). Both angles are measured with respect to the DICOM LPS axes, effectively aligning with the patient’s anatomical orientation.

Catering to the large variability of the electrical axes, a cutoff was applied to the vectors more than 3 SD from the population mean, which was less than 3% of the sample. Finally, two metrics were used to quantify the mutual alignment between the 20 pairs of anatomical-electrical definitions of axes, as described next.

#### 2.6.1. Angular measurements.

For each method pair, the angle between each anatomical and electrical axis, $\Delta AE_{3D}$, was measured across the cohort, providing information on the extent of spatial separation between the two vectors. $\Delta AE_{3D}$ was computed as: $\;\Delta AE_{3D}\;=\;\cos^{-1}\left(v_{anatomical}\cdot v_{electrical}\right)$. The error metric, termed as *Spatial consistency*, is inversely related to the standard deviation of $\Delta AE_{3D}$ across the population, with higher spatial consistency indicating a lower standard deviation. Since the angle measured is the smallest angular difference in 3D, it does not capture values that account for differences in the orthogonal planes. Therefore, angular differences were also calculated in the three anatomical planes and using the spherical coordinate measures, (θ, ϕ), to obtain Δθ, Δϕ. This approach made the angular differences direction-dependant, enabling a more detailed examination which incorporates planar measures. The angular metrics were fitted to a normal distribution to quantify their spread, and the mean axes were computed and plotted for the lowest spatial consistency pair.

#### 2.6.2. Geodesic distances.

The mean geodesic distance (MGD) between vectors (i.e., axes) was chosen as the error metric due to its suitability for quantifying angular differences between unit vectors on a sphere. The anatomical-electrical relationship was studied across all method pairs by measuring the MGD between the unitary directional vectors of anatomical and electrical axes after their population-based rotational alignment. In other words, the average rotational relationship between both axes is learned and used to infer the electrical axis from the anatomical axis – the smaller the residual (i.e., the MGD) of that inference, the strongest the anatomical-electrical axes relationship.

A population-based rotation matrix T was computed to map anatomical to electrical vectors using an 80/20 train/test split of the healthy cohort. T was computed used singular value decomposition (SVD) of the correlation matrix, H = E_vectors * A_vectors, where E_vectors and A_vectors represent the matrices of electrical and anatomical unit vectors, respectively. The rotation matrix T was then computed as T = U * V′, where U and V are the left and right singular vectors of H. This process was repeated for each of the 20 method pairs (4 electrical × 5 anatomical methods). For each method pair, MGDs were analysed between predicted and true electrical vectors. The performance of each method pair was evaluated on the unseen test set, with lower MGD values indicating better prediction of the electrical vectors. A heatmap was used to visualize trends and absolute MGD values for each pair of axes.

Note that the rotation matrix (T) and the associated MGD were employed solely to identify the best definitions of the anatomical and electrical axes, while our main results are derived directly from the untransformed axes.

### 2.7. Characterisation in healthy population

#### 2.7.1. Distribution analysis.

The derived angular metrics were analysed using a bivariate approach to characterise the distribution of the anatomical and electrical spherical coordinates. Kernel density estimation was employed to generate contour plots for both the individual axes and their differences, while the marginal distributions were modelled using a normal distribution. Pearson’s correlation coefficient (r) was calculated to quantify the relationship between the anatomical and electrical spherical metrics, and scatter density contours were used for visualisation.

#### 2.7.2. Demographic analysis.

BMI and age were first discretised into four subcategories and then further stratified by sex. Violin plots were used to visually represent the distributions of the anatomical and electrical spherical coordinates, as well as their differences, by integrating kernel density estimates with descriptive measures. Statistical comparisons subsequently identified significant differences in angular metrics across the stratified groups, and multivariable linear regression was conducted as detailed in Section 2.9.

### 2.8. Primary hypertension cohort

The ICD-10 diagnosis code I10 for *primary hypertension* was used to identify the cohort. 3,568 subjects were identified from the hypertensive cohort with primary hypertension diagnoses as the *only* cardiovascular diagnosis, hence excluding the effect of other structural or functional pathologies. Next, differences in the distributions of the spherical coordinates of anatomical-electrical axes were investigated using the methods proposed as best-performing (Section 2.6). The bivariate distributions of the anatomical–electrical spherical coordinates in healthy and hypertensive cohorts were compared using kernel density estimates and box plots, with pairwise statistical testing. Separately, multivariable linear regression analysis (detailed in Section 2.9) was used to assess the impact of demographic factors and pressure metrics on anatomical orientation.

### 2.9. Statistical analysis

BMI and body surface area (BSA) values were computed using height and weight measurements during the visit where the cMRI and ECG were acquired. BSA was calculated using the Du Bois formula [27]. Subjects with missing data for BMI or BSA were excluded of the respective analysis. For the healthy cohort (N = 27,832), missing data included 783 for BMI and BSA and 4,130 for pulse wave analysis, while the hypertensive cohort (N = 3,568) included 91 missing for BMI and 517 for pulse wave analysis. Sex categorisation was binary (male/female) as acquired from the NHS registry during volunteer recruitment.

Levene’s test was applied to assess the homogeneity of variances (homoscedasticity) in the angular distributions, centring on the mean. Two-tailed Mann-Whitney U test was used for testing sex differences within BMI and age groups, as well as healthy vs hypertensive groups. Additionally, pairwise Mann-Whitney U tests were conducted between all combinations of the four BMI and age groups to assess differences in angle. p < 0.001 was considered statistically significant. The choice of Mann-Whitney U tests is due their non-parametric nature, making minimal assumptions about data distributions.

Multivariable linear regression analyses were conducted to allow simultaneous evaluation and adjustment for multiple covariates. A multivariable linear regression was conducted to examine the effect of BMI, age, and sex on the angular metrics of the cardiac axes, where the angular measures were the response variables. In the study of the impact of hypertension, a second multivariable linear regression was conducted to assess the association of different parameters on the anatomical orientation in both healthy and hypertensive cohorts, constrained to the frontal plane. The response variable was $\theta_{Anatomical}$, and the predictor variables included age, sex, BMI, BSA, mean arterial pressure (MAP), and arterial stiffness (AS). Prior to analysis, values were standardised to remove the effect of different measurement scales, thereby allowing a direct comparison of the impact strength of each predictor on the anatomical frontal orientation. This is equivalent to standardised regression coefficient of $\beta_{i}^{*}=\beta_{i}/{SD(X}_{i})$, where ${SD(X}_{i})$ is the standard deviation of predictor variable i. The p-values and confidence intervals for these coefficients were used to identify which predictors had statistically significant effects. Multicollinearity among predictor variables was assessed using pairwise correlations and variance inflation factor (VIF) with a threshold of <5 considered acceptable [28].

Two phenome-wide association studies (PheWAS) studies were conducted using the UK BioBank data and the derived axes metrics. For both studies, potential confounders — age, sex, sex-age interaction (sex*age), height, and weight — were regressed out. Phenotype data were normalised and cleaned by discarding columns where 95% of the data were identical. Univariate cross correlations were computed between each of the 6 de-confounded axis metrics and the normalised phenotypes. Pearson correlation was used for the first PheWAS due to the phenotypes being continuous, while Spearman correlation was used for the diagnoses as they were binary.

The first PheWAS followed a similar approach to [29], and employed data from the healthy cohort (N = 27,832). Eight relevant categories were identified including demographics (1001), early life (1002), abdominal MRI (1016), physical measures (1006), 12-lead ECG at rest (104), LV size and function (133), pulse wave analysis (128), and cardiac and aortic function (157). Where two phenotypes had a correlation coefficient >0.9999, one was removed. The second PheWAS followed a methodology similar to [30]. UK BioBank diagnoses, originally in ICD-10 codes format, were converted to phecodes using a PheWAS Catalog mapping, with diagnostic groups identified [31]. Both prevalent and incident diagnoses were used in this exploratory analysis.

## 3. Results

A total of 39,123 participants passed ECG quality filtering and had paired cMRI and ECG data. Among them, 27,832 were classified as healthy.

### 3.1. Characteristics of the healthy population

This cohort had a mean age of 63.3 ± 7.7 years, a mean BMI of 25.8 ± 4.2 kg/m^2^, and a sex split of 56% female and 44% male. These subjects had no history of cardiovascular disease at the time of assessment and for up to one year afterward. Further baseline characteristics are included in Table 2.

**Table 2:** Healthy cohort’s demographic and clinical baseline characteristics, N = 27,832. Data is presented as mean ± standard deviation or N (percentage). BSA = Body surface area, LVEF = Left ventricular ejection fraction, LVEDV = left ventricular end diastolic volume.

Metric	Healthy cohort N = 27, 832
Sex - Female	15,625 (56.1%)
Sex - Male	12,207 (43.9%)
Age	63.3 ± 7.7
Ethnicity - White	26,859 (96.5%)
Ethnicity - Other	973 (3.5%)
BMI (kg/m^2^)	25.8 ± 4.2
BSA (m^2^)	1.85 ± 0.2
QRS duration (ms)	87.0 ± 12.5
LV mass (g)	83.3 ± 20.9
^*^LVEF (%)	56 ± 6.0
^*^LVEDV (mL)	137.5 ± 59.2

* Values from Siemens *syngo* InlineVF (Siemens Healthcare, Erlangen, Germany).

### 3.2. Definition of anatomical and electrical axes

The criteria for maximising the mutual alignment between the electrical and anatomical axes led to their optimal definitions: the anatomical axis was defined as the vector from the apex to the spatial centre of the four valves (VPA), and the electrical axis as the direction of the maximum QRS dipole (maxQRS). This selection was guided by two metrics,angular distributions and rotation residuals, as detailed in the following sections.

In the present study, the Kors transform was used to construct the VCG from the 12-lead ECG instead of alternative methods, such as the inverse Dower transform or the Q Least Squares Value (QLSV) transform. This choice was taken because comparative studies consistently demonstrate that the Kors transform provides the closest approximation to the Frank VCG, outperforming the Dower method and exhibiting similar or superior accuracy compared to QLSV [32,33]. While subtle differences in vector orientation might occur with other transforms, these are expected to be modest and unlikely to influence the results of this study.

#### 3.2.1. Angular distributions.

**Fig 4:**
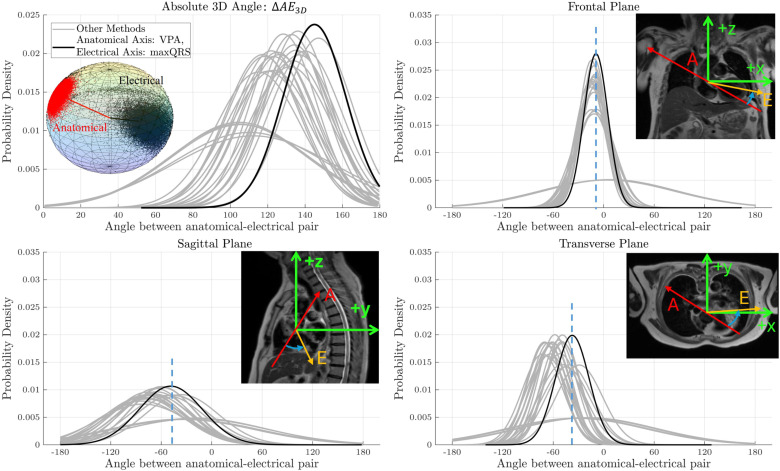
Angular distributions of the anatomical-electrical angular differences in 3D and in the three anatomical planes. Blue arrows show the angle portrayed in the corresponding plot (ACW is negative). The dashed lines correspond to the average separation between the mean anatomical (red arrow) and electrical (orange arrow) axes in each plane. The narrower spread (lower standard deviation) corresponds to a higher spatial consistency, i.e., a more consistent anatomical-electrical relationship across the population. Note that acute angles were used to avoid artificial extremes due to the + 180/-180 boundary.

Fig 4 shows the angular distributions across the whole population for all anatomical-electrical definition pairs. The highest spatial consistency, i.e., lowest SD, was exhibited by the VPA anatomical axis and maxQRS electrical axis. In 3D space, the cosine SD of this pair was 0.19, compared to 0.60 for the least spatially consistent pair. This highlights the wide range of spatial consistencies observed. The most visible difference and lowest SD is in the frontal plane, and the VPA- maxQRS pair maintains its property of having the highest spatial consistency across all three planes.

The difference in variances among the distributions was assessed using Levene’s test, confirming that all variances were significantly different from each other.

#### 3.2.2. Rotation residuals.

**Fig 5:**
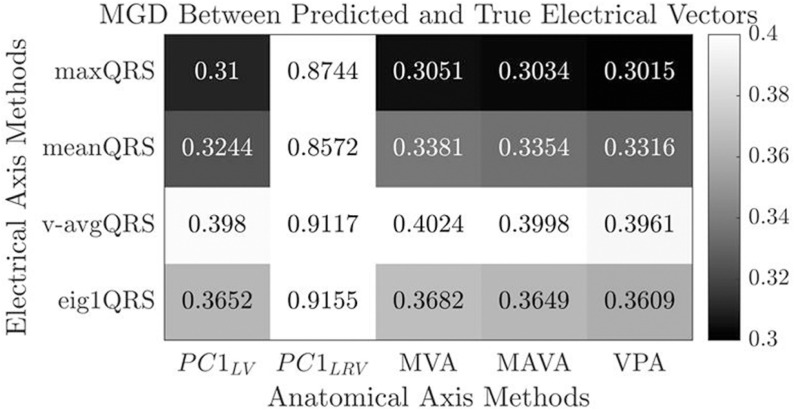
Heatmap of the mean geodesic distance between predicted and true electrical axes for all anatomical-electrical pairs.

In order to identify the method pair that best captures the anatomical-electrical interplay, the mean geodesic distance between the true electrical vectors and those predicted from the anatomical axes was computed using a derived rotation matrix, as depicted in Fig 5. A stronger linear relationship between each anatomical-electrical definition pair is associated with a lower error, indicated by a more robust transformation matrix. The error magnitude varied between 0.92 and 0.30, corresponding to the pairs: ${PC1}_{LRV}$ - eig1QRS and VPA & maxQRS respectively. The combination of maxQRS and VPA emerged as the optimal pair, achieving the most accurate prediction of electrical vectors. There is a notable reduction in error when the right ventricle is incorporated into the calculation of the anatomical axis, regardless of the electrical axis method used.

#### 3.2.3. Optimal definition of anatomical and electrical axes.

Following evaluation of all 20 pairs, the analysis identified VPA-maxQRS as the best-performing pair for both spatial consistency and transformation residuals. This finding reinforces VPA as a reliable definition of the anatomical axis which includes the whole biventricular geometry. Additionally, maxQRS proves to be a robust, simple method for defining the electrical axis. The mean angular separation ($\Delta AE_{3D}$,) was 145.0° ± 16.8°, with median [IQR] of 146.7° [18.8°].

### 3.3. Distribution and interplay of cardiac axes in healthy population

**Fig 6:**
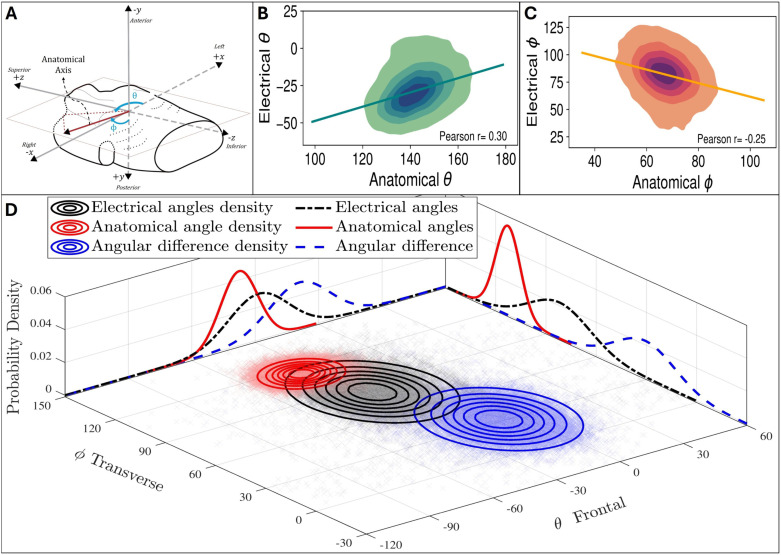
Distribution analysis of the anatomical and electrical axis orientation metrics. **(A)** Torso diagram with spherical coordinates (θ, ϕ) for an example cardiac anatomical axis, where ϕ represents the extent to which the base is pointing to anterior or posterior. **(B)**(C) Contour plots with regression line and Pearson r, showing the anatomical-electrical linear relationship of θ and ϕ. **(D)** Bivariate (θ, ϕ) probability density distributions of electrical and anatomical angles, and their differences (normal-fitted marginal distributions and kernel density estimation contours) indicating complex interplay between the two axes. (The anatomical axis presented here is inverted to allow ease of visualisation.).

A bivariate distribution analysis was performed using the anatomical and electrical spherical coordinates, along with their angular differences, as shown in Fig 6. There is a markedly narrower spread of the anatomical axes, which is spatially illustrated in Fig 6D and quantified from the marginal distributions. Additionally, the underlying distributions (from density estimates) show the electrical axis as the primary factor influencing the variability in the angular difference. The constrained angular difference distribution suggests a discernible yet secondary impact from the anatomical axis, indicating a degree of anatomical-electrical coupling. Fig 6B and 6C shows moderate correlation between anatomical and electrical θ and ϕ.

### 3.4. Demographic analysis

**Fig 7:**
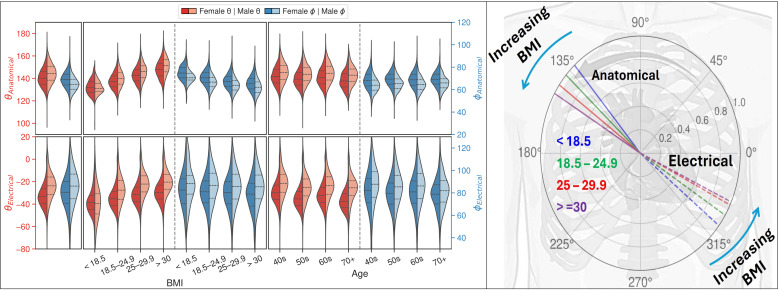
Violin plot representations of $\theta_{Anatomical}$, $\theta_{Electrical}$, $\phi_{Anatomical}$, $\phi_{Electrical}$ divided by BMI and age groups along with their respective sex distributions. Left panel shows the distributions for the entire population. Increasing BMI is accompanied by an increase in $\theta_{Anatomical}$ and $\theta_{Electrical}$. Shifts in angle between sexes are significant at p < 0.001 for all groups except BMI < 18.5.

Fig 7 provides a comprehensive illustration of the association between BMI, age, and sex and the anatomical-electrical angular measures. For distributions of Δθ and Δϕ, see Fig C in S1 Appendix.

BMI is the parameter with the strongest impact on anatomical orientation in the frontal plane ($\theta_{Anatomical}$), with a more horizontal orientation as BMI increases. The change seen in $\theta_{Anatomical}$ is in the same direction as $\theta_{Electrical}$, suggesting anatomical-electrical coupling. Age has little to no visible impact on either orientation measures. Pairwise comparisons at p < 0.001 revealed significant differences between all combinations of BMI and age groups for $\theta_{Anatomical}$, $\theta_{Electrical}$ and $\phi_{Anatomical}$, but only age for $\phi_{Electrical}$. The distributions also showed that male hearts shift in the same direction as those reported by an increase in BMI. These differences were statistically significant at p < 0.001 for all groups except BMI < 18.5. Univariate Pearson correlations align with these observations, as seen in Table F in S1 Appendix.

**Fig 8:**
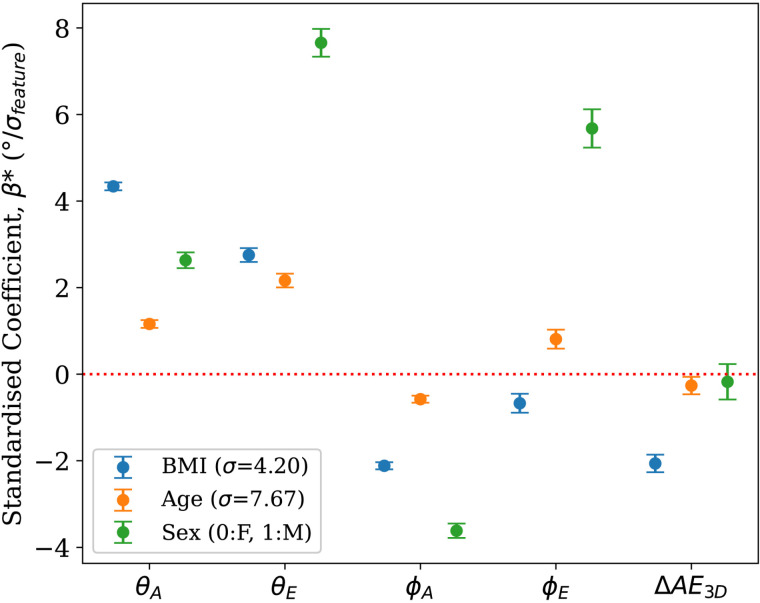
Regression coefficients illustrating the relative influence of BMI, age, and sex on cardiac anatomical and electrical axes. The standardised coefficient, $\beta^{*}$, indicates the change in the angle per standard deviation of the predictor. Absolute values of the coefficients represent the strength of impact each variable has on the angular metrics. Note that coefficient of sex is not standardised by standard deviation and is for Male compared to Female. All multicollinearity and goodness-of-fit results can be found in Tables A–E in S1 Appendix.

The regression analysis, shown in Fig 8, reinforces these patterns while accounting for covariates. Negligible multicollinearity was found with predictor pairwise correlations of |r| < 0.12 and VIFs < 5. BMI demonstrates a strong impact on anatomical orientation in the frontal plane ($\theta_{Anatomical}$). This effect is paralleled, though slightly weaker, in the electrical orientation ($\theta_{Electrical}$), signifying robust anatomical-electrical coupling. BMI exhibits moderate negative effects on anatomical orientation in the transverse plane ($\phi_{Anatomical}$), with a weaker negative influence on electrical orientation ($\phi_{Electrical}$). Age shows a slight positive effect on $\theta_{Anatomical}$ and a moderate effect on $\theta_{Electrical}$, both in the same direction. However, the impact of age on transverse plane measures is discordant, with a minimal negative effect on $\phi_{Anatomical}$ and a slight positive effect on $\phi_{Electrical}$. Sex differences are most pronounced in the electrical axis, with males showing strong positive effects on both $\theta_{Electrical}$ and $\phi_{Electrical}$. The effect on the anatomical axis is much weaker for $\theta_{Anatomical}$ and shows a moderate negative effect for $\phi_{Anatomical}$. The opposing directions for ϕ coefficients suggest that intrinsic sex-differences outweigh anatomical-electrical coupling in this plane. Lastly, BMI was the only significant factor influencing $\Delta AE_{3D}$, possibly due to the lower sensitivity of the electrical axis to changes in BMI compared to the anatomical axis, while age and sex showed no significant impact.

### 3.5. Primary hypertension cohort

**Fig 9:**
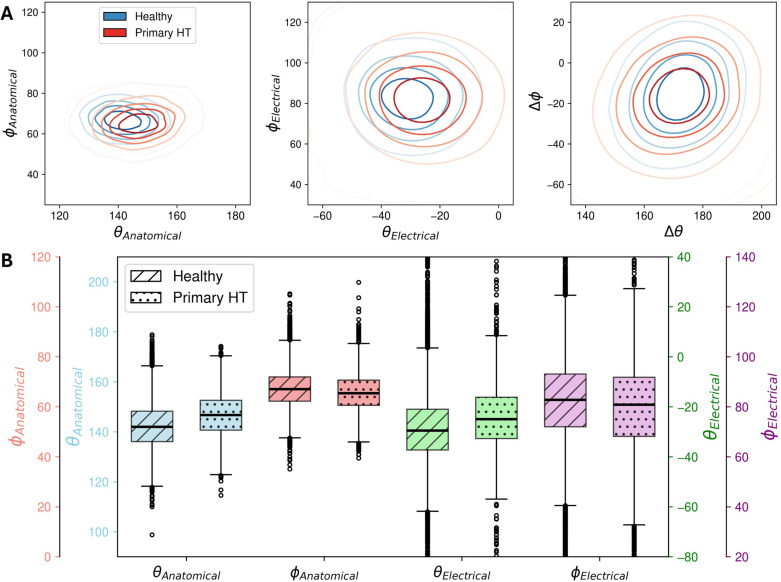
Effect of primary hypertension on the cardiac anatomical and electrical axes. **(A)** Kernel density estimates for the comparison of axes angular measures in healthy subjects and subjects with primary hypertension. “x” markers represent the median of each group **(B)** Box plot comparison of the metrics. All results were significantly different between the two groups at p < 0.001. Healthy N = 27,832; Primary hypertension N = 3,568.

There was significant difference in all measures ($\theta_{Anatomical}$, $\theta_{Electrical}$, $\emptyset_{Anatomical}$, $\emptyset_{Electrical}$) between the healthy and hypertensive cohorts, at p < 0.001, see Fig 9. A clear shift occurs in the anatomical axis that is replicated, albeit to a lesser extent, in the electrical axis distributions. Despite changes in anatomical and electrical axes, the relationship (Δθ and Δϕ) between them remains relatively stable. This observation is maintained even within each BMI group, shown in Fig D in S1 Appendix. The lack of a significant shift in the angular difference distributions (Δθ and Δϕ), in addition to the unidirectional shift for both axes, is indicative of anatomical-electrical coupling. Angular separation, $\Delta AE_{3D}$, differed significantly between healthy (145.0° ± 16.8°) and disease (137.8° ± 27.5°) groups at p < 0.001. Levene’s test also confirmed significantly greater variability in the disease group at p < 0.001. These findings indicate that while anatomical-electrical coupling persists in disease, it is attenuated by pathological influences.

**Fig 10:**
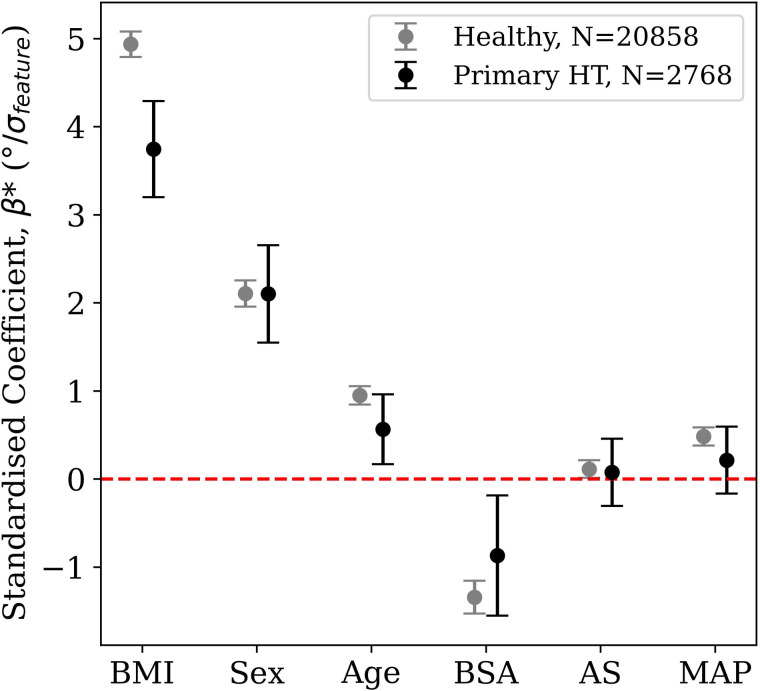
Multivariable linear regression results comparing healthy and primary hypertension cohorts (Healthy, R^2^ = 0.31; Primary hypertension, R^2^ = 0.18). Standardised coefficients, $\beta^{*}$, with 95% confidence intervals for each predictor, represent strength of impact of each independent variable on $\theta_{Anatomical}$. BSA (Body Surface Area), AS (arterial stiffness), and MAP (mean arterial pressure). All multicollinearity and goodness-of-fit results can be found in S1.4 in S1 Appendix.

The factors that predict $\theta_{Anatomical}$ differed between the healthy and hypertensive cohorts. The regression coefficients that allow direct comparison of the variable impact strength on each parameter are presented in Fig 10, with VIF < 5 for all variables confirming negligible collinearity affecting the regression. They show how the effect of BMI and BSA on $\theta_{Anatomical}$ is reduced in the hypertensive group.

In terms of the metrics related to an increased afterload, it was confirmed that the hypertensive group had higher CSBP, MAP and AS at p < 0.001 (Fig E in S1 Appendix). MAP and AS showed no statistically significant effects on $\theta_{Anatomical}$ in either group. Wider confidence intervals observed in the hypertensive group can primarily be attributed to the smaller sample size (N = 2,768) compared to the healthy group (N = 20,858). Additionally, the increased variability in the data may suggest more heterogeneity in how the investigated parameters affect $\theta_{Anatomical}$, which is highlighted by the reduction of R^2^ from 0.31 in the healthy cohort to 0.18 in the hypertensive cohort.

### 3.6. Phenome-wide association study

**Fig 11:**
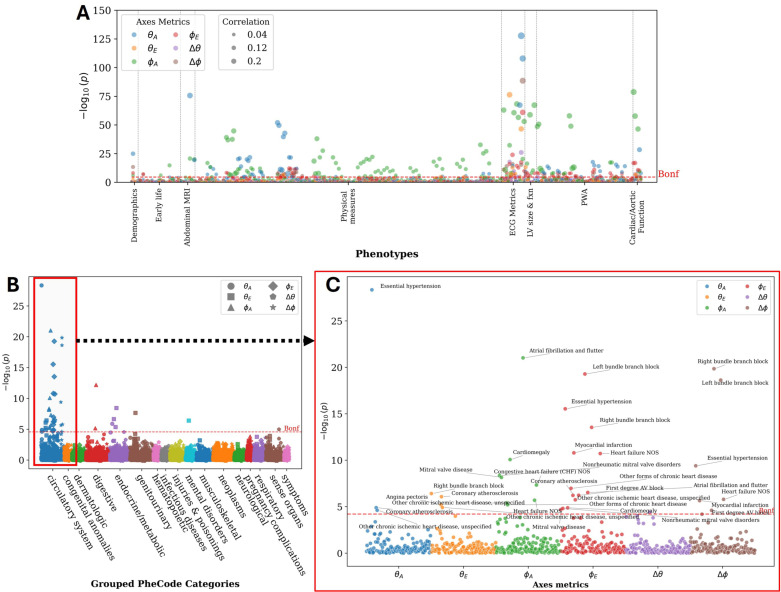
Manhattan plots of the p-values for correlations between the angular metrics of cardiac anatomical-electrical axes and (A) UK BioBank phenotypes, (B) clinical diagnoses, and (C) breakdown of cardiovascular diagnoses associations. P-values are from a two-sided t-test.

The Manhattan plot in Fig 11 shows the p-values for correlations between the six axis metrics and 350 phenotypes, with 274 significant associations crossing the Bonferroni threshold. $\theta_{Anatomical}$ was notably correlated with the 12-lead ECG axes (T axis, R axis, and P axis), as well as with visceral adipose tissue volume, diastolic blood pressure and ascending aorta distensibility. Meanwhile, $\phi_{Anatomical}$ demonstrated significant associations with left ventricular end-diastolic and systolic volumes (LVEDV and LVESV) and ventricular rate, alongside associations with QT interval, and LV stroke volume. $\theta_{electrical}$ and $\phi_{electrical\;}$ were primarily linked to ECG measures like QRS duration and PQ interval, indicating a relationship with cardiac conduction times. These electrical axis metrics were also associated with blood pressure measurements, as well as structural and functional heart metrics like LVESV, LVEDV, and left ventricular ejection fraction (LVEF). Table G in S1 Appendix details the top associations for each axis metric.

The relationship between clinical diagnoses and the six axis metrics was explored through the PheWAS in Fig 11. Conduction disorders, particularly bundle branch and fascicular blocks, were most significantly associated with and , introducing potential relevance of the electrical axis in the non-frontal planes. Primary (Essential) hypertension was also significantly associated with , and . Other notable associations are that of atrial fibrillation and mitral valve disorders with . Furthermore, associations with non-cardiac diagnosis phenotypes, included diaphragmatic hernia with . Table H in S1 Appendix reports all the significantly associated clinical diagnoses and their correlations.

## 4. Discussion

In this study, we performed a population-wide investigation of cardiac anatomical and electrical axes using paired cMRIs and ECGs. By maximising the mutual alignment between anatomical and electrical axes across 28,000 healthy subjects, we proposed standard axes definitions. We then characterised anatomical-electrical coupling in healthy conditions and primary hypertension. Findings demonstrated that a more horizontal electrical axis, linked to a more horizontal cardiac orientation, was associated with increasing BMI, and in males compared to females. The anatomical axis was primarily influenced by BMI, followed by sex, with minimal contribution from age. The transverse plane revealed distinct sex-based differences in the axes. In the hypertensive cohort, where cardiac orientation showed a similar shift, the electrical axis mirrored this change, emphasising the strong coupling.

Understanding the anatomical-electrical coupling and its interaction with patient-specific factors, like BMI and sex, demonstrates how traditional population-based interpretations have the potential to evolve into more patient-centric approaches, such as higher degrees of personalisation in clinical applications like ECG imaging. The findings support the development of personalised ECG interpretation criteria that could adapt to individual characteristics, further contributing to the clinical utility multimodal, patient-specific data.

### 4.1. Proposed definitions of cardiac axes

For each electrical axis definition, the performance of VPA as the anatomical axis consistently surpassed those of the four other definitions across both metrics of mutual alignment. Using specific anatomical landmarks ensures physiological anchoring to cardiac anatomical structures, explaining this robust performance. The contrasting, inferior performance of the biventricular axis of inertia can be attributed to the globular nature of the respective 3D point clouds.

The anatomical axis definitions were validated against previously published expert manual measurements derived from cMRI, which defined the axis from the LV apex to the mitral annulus (frontal: 38° ± 10°, transverse: 46° ± 7°) [4]. Close agreement was found using identical landmarks (MVA; frontal: 39.1° ± 12°, transverse: 49° ± 9°), with minor transverse-plane differences expected from our chosen basal landmark (VPA; frontal: 37.4° ± 9°, transverse: 29.5° ± 9°). This highlights the robustness and consistency of our automated three-dimensional definitions.

Assessing the electrical axis, we found that maxQRS had optimal alignment with the anatomical axis, in both spatial consistency (Fig 4) and rotational residuals (Fig 5). This definition excels in capturing the anatomical-electrical alignment as it is an effective representation of the direction of propagation of the electrical wavefront [34]. An additional practical advantage is that maxQRS relies on the identification of a single time point, independent of the pre-defined QRS start and end points, enhancing measurement robustness and reliability.

### 4.2. Anatomical-electrical axes variability and coupling

The greater degree of electrical variability can be explained by the fact that anatomical variability is inherently included within electrical variability, whereas anatomical variability is solely structural. This larger variance is also in agreement with the fact that the QRS segment is influenced by a more diverse set of physiological mechanisms and acquisition aspects, including but not limited to: (i) fast conduction system defined by the Purkinje fibre network; (ii) cardiac geometry factors beyond orientation, such as variability in myocardial thickness [35]; iii) variability in the microstructure, such as the presence of fibrosis and (iv) acquisition-related variables, such as the spatial placement of electrodes. Anatomical structures affecting depolarisation, such as myocardial volume, also exhibit considerable inter-individual variability [36]. Additionally, the high control and precision of a cMRI allows consistent capturing of the heart-torso orientation. Another aspect of consideration is the heart’s interaction with the pericardium and its surroundings. The fibrous pericardial sac’s attachment to the diaphragm tendon and the dorsal sternum via ligaments are driving factors in its limited movement in the mediastinum [37].

Results support a considerable degree of coupling between anatomical and electrical axes. The moderate correlations observed between anatomical and electrical angles (Fig 6) align with previous research, but with a slightly stronger correlation (r = 0.3 compared to r = 0.23 reported in [4] for the frontal plane angle). The fact that the distribution of angular differences is not wider than the distribution of electrical axes is a second finding that illustrates the axes coupling. Studying the factors that distort the coupling between these two axes is proposed as a novel way to investigate the impact of cardiovascular diseases.

### 4.3. Demographics and cardiac axes

BMI, age, and sex influence both axes with different strengths and direction of change, suggesting the presence of several contributing mechanisms.

***BMI-related effects*:** The 3D angular separation, $\Delta AE_{3D},$ was only significantly influenced by BMI as illustrated in Fig 8, which could be a result of different anatomical and electrical remodelling interactions coupled with the pure rotation of the anatomical axis. Cardiac remodelling, from increased epicardial adipose tissue, involves molecular-level changes in structural and ionic properties [38,39], thus probably affecting the electrical axis differently.

BMI was the factor with the strongest influence on the anatomical axis (β = 4.3 and β = -2.1 in the frontal and transverse planes, respectively). The increase in BMI resulted in rotation of the heart to a more horizontal direction, where the base is becoming more inferior and slightly more posterior. This rotation translates to the apex moving closer to the chest wall and pointing more to the left. The observed changes could be attributed to factors associated with increased BMI: (i) enlarged liver exerting an upward force on the apex; (ii) reduced diaphragm contractility due to thickening and fibrosis; (iii) increased left ventricular mass; and (iv) biventricular remodelling [40–43].

***Age-related effects*:** Although the overall impact of age was minimal compared to BMI and sex, age still contributed to changes in cardiac axes. In the frontal plane, a modest positive effect of age was observed on the angular metrics, while in the transverse plane, age exerted only minor, oppositely directed effects. These subtle age-related changes suggest that, beyond the anatomical remodelling driven by BMI and sex, age may influence the electrical axis through additional, yet less pronounced mechanisms.

***Sex-related effects*:** Males generally exhibited more horizontally oriented hearts compared to females. Notably, the effect of sex on $\theta_{Electrical}$ (β** = 7.7**) was three times greater than on $\theta_{Anatomical}$ (β = 2.6), with a similar magnitude trend in the transverse plane. Established sex differences in cardiac structure, such as lower ventricular mass in females [44], might contribute to the observations. Additionally, sex-specific variations in cardiac electrophysiology, such as differences in action potential duration, calcium ion release and reuptake rates, and myocardial contraction and relaxation velocities, have been documented in previous studies [45,46]. The combination of changes in heart geometry and sex-specific ion channel activity [47], along with sex-specific variations with age [45] could be playing a more significant role in the effect on the electrical axis than the anatomical axis, particularly in the transverse plane. Thus, ECG interpretation in certain leads may be more affected than others by patient-specific variations.

***Anatomical-electrical interaction*:** In the frontal plane, the electrical and anatomical axes shifted in the same direction under the effect of all three demographic factors plane (Fig 8). A change in the signal acquired from the electrodes can be a direct result of the change in the solid angles between the heart and the electrodes [48], partially explaining the observed rotation of the electrical axis in the same direction as the anatomical as BMI increases or both axes being more horizontal in males.

The transverse plane displayed different degrees of discordance between the anatomical and electrical shifts. BMI had minimal effect on the electrical transverse angle, sex changed both axes but in opposite directions and age displayed a smaller effect again in opposite directions. This suggests that other intrinsic factors may have a dominant effect in the shift of the electrical axis in this transverse plane.

### 4.4. Primary hypertension affects cardiac axes and their interplay

Whether due to myocardial infarction or changes in afterload and preload, cardiac remodelling occurs as a result of physiological and pathological changes [49–51]. This investigation of the effect of primary hypertension on anatomical and electrical axes additionally highlights orientation changes and highlights a potential new sign of remodelling with promising diagnostic or prognostic value.

Results demonstrate that hypertension is linked with a significant shift in anatomical axes that mirrors the pattern observed with increasing BMI — namely, a more horizontal orientation (Fig 9). This rotational change aligns with left ventricular axis shifting seen during remodelling in aortic stenosis [52], also a condition associated with increased afterload. The distributions show a concurrent shift of the electrical axes in the same direction, providing additional evidence of sustained anatomical-electrical coupling in hypertension. The manifestation of this coupling is also illustrated by the small shift of the angular difference distributions.

Moreover, the significantly greater variability of $\Delta AE_{3D}$ in the disease group may indicate a weakening of this coupling, where pathological influences begin to disturb the anatomical-electrical relationship seen in healthy hearts. A reason for this phenomenon could be the combination of both anatomical and electrical remodelling. Ample evidence supports the occurrence of both types of remodelling in response to increased afterload, with electrical changes sometimes preceding anatomical alterations in left ventricular hypertrophy [53,54].

In the search for the factors that explain the anatomical cardiac orientation, differences between the healthy and hypertensive cohorts were found: hypertension was associated with a reduced magnitude of the BMI and BSA coefficients of the regression model (see Fig 10), and an increase of the metrics of afterload (CSBP, AS, MAP). This finding suggests that the underlying pathology (primary hypertension) is contributing to the change in the anatomical axis. Although PWA metrics were elevated in the hypertensive group, none showed a significant effect, indicating that these factors are unlikely to be the primary drivers of the anatomical changes observed in the hypertensive group.

### 4.5. Phenome-wide association study

The PheWAS approach in this study is primarily aimed at hypothesis generation, and while the associations found have low correlation coefficients, their statistical significance suggests avenues for future research. For instance, the association between $\theta_{Anatomical}$ and aorta distensibility raises questions about the impact of vascular changes and subsequent remodelling on anatomical orientation, a mechanistic link that has been hypothesized to explain the change of orientation in aortic stenosis [52].

Results also reinforce existing observations, but also introduce new hypotheses about how pathological and physiological remodelling may affect ECG morphology. Multiple statistically significant associations were found between axes metrics and clinical diagnoses, where the metrics in the transverse plane and of the electrical axis were the ones with more associations found. Only the electrical metrics were linked to conduction disorders as would be expected, e.g., bundle branch blocks. The link to atrial fibrillation and flutter is in line with prior studies that have associated the LV sphericity index with this diagnosis group, as noted in [30].

### 4.6. Clinical relevance and implications

Current clinical approaches to 12-lead ECG interpretation leverage a tiny fraction of the data they contain and are limited by uncertainty with regard to the clinical implications of as-yet uncharacterised abnormalities that are present [55]. Strategies to increase the diagnostic accuracy of ECGs are therefore of great clinical interest and include the identification and characterisation of new biomarkers which may support early or more accurate identification of pathology in a widely used, rapid and safe test [56–58].

Our demonstration that anatomical-electrical axes change in the context of hypertension indicates the possibility of a novel measure derived from ECG or imaging that could be used for screening for this condition or its complications. If this biomarker were demonstrated to be of predictive value for the development of this condition or its complications, then ECG identified anatomical-electrical shifts during routine ECG screening could identify individuals at risk of developing cardiovascular pathologies, prompting timely clinical follow-up. For example, a situation is envisaged in which a patient with hypertension showing progressive electric or anatomical axis deviation despite medical therapy might undergo reassessment of blood pressure control, uptitration of medical therapy or earlier echocardiographic assessment for ventricular remodelling. Similarly, a significant change in electrical axis without a corresponding change in BMI —ruling out anatomical variability— could be an early indicator of changes in conduction patterns. It is emphasised that these scenarios are purely hypothetical but are presented to demonstrate possible uses for rigorously assessed biomarkers.

Previous reports have demonstrated the diagnostic value of the cardiac anatomical axes, exemplified by axis shifts predicting cardiac mass regression in aortic stenosis [52]. Through integration into automated ECG or cMRI software, the standardised anatomical and electrical axis definitions proposed in this study could serve as biomarkers, offering a personalised insight, such as angular metrics in standard ECG reports alongside traditional measurements but augmented by patient-specific factors.

Demographic influences on cardiac axes have significant implications for ECG interpretation [59,60]. Quantifying these variations could enable demographic-specific reference ranges, reducing systematic biases in ECG analysis across diverse populations. The characterisation of these changes will promote accurate personalised ECG interpretation. For instance, patients with elevated BMI may exhibit anatomical orientations that would result in false positive or negative using standard ECG criteria, where a more patient-centric approach could avoid that.

Finally, our results identify further opportunities of research using electrophysiological simulations to investigate the mechanisms of how anatomical variability influences the VCG/ECG signal – note that all computational meshes and results are made available through the UK BioBank.

### 4.7. Limitations

The study’s use of automatically generated reconstructions of cardiac biventricular geometry may have omitted some anatomical details, though this is unlikely to significantly impact the results due to the global anatomical regions used in axis definition.

The UK Biobank population is known to exhibit a “healthy volunteer bias”, where the population is has a lower risk factor profile and lower all-cause mortality [61,62]. While this may not significantly affect our investigation of anatomical-electrical coupling in a healthy cohort, future research on pathological populations should draw from diverse databases to ensure valid applicability. Additionally, the study population has overwhelming majority (96%) of white ethnicity. Certain trends and associations may be different in other ethnic groups, particularly if genetic factors influence individual axes or their coupling.

Furthermore, representation of inter- and intra-individual variability could be further built upon from our framework. Previous work highlighted the significance of computational methods for exploring the electrophysiology variability [63], which results from heterogeneity in ionic, anatomical, and structural parameters. Exploring parameter diversity can identify subgroups at particular risk for arrhythmias or conduction disorders [64,65]. Hence, capturing and representing the range of variability play important roles in the applicability of cardiac digital twins in personalised medicine. Whether it is customising parameters from a patient or building a cohort of digital twins to represent a spectrum of variation, the incorporation of population-level data facilitates patient-centric scientific insights, especially when results hold across diverse populations.

## 5. Conclusion

This study introduces standard definitions for cardiac anatomical and electrical axes based on 3D alignment criteria from a population of cMRIs and ECGs. Complex anatomical-electrical coupling is demonstrated by the varying magnitude and direction of associations. Cardiac axes change in the presence of cardiovascular conditions, but so does the extent of anatomical-electrical interplay, indicating altered coupling in disease. Future work will include further exploration of cardiac axes interplay and the influence of pathology using comprehensive, dynamic cardiac digital twins, with the aim of developing personalised ECG interpretation that accounts for individual anatomical and pathological characteristics.

## Supporting information

S1 Appendix PDF of Sections S1.1-S1.5 for “Anatomical-electrical coupling of cardiac axes: definitions and population variability for advancing personalised ECG interpretation”. (DOCX)
