# Supplementary material for: Anatomical-electrical coupling of cardiac axes: Definitions and population variability for advancing personalised ECG interpretation
Source: PLoS Comput Biol. 2025 Jul 10;21(7):e1013161. doi: 10.1371/journal.pcbi.1013161 (PMC12244483; doi:10.1371/journal.pcbi.1013161)
Supplement: S1 Appendix — (DOCX) [file pcbi.1013161.s001.docx]

**S1 Appendix**

Anatomical-electrical coupling of cardiac axes:
definitions and population variability for advancing personalised ECG interpretation

**S1.1. Surface mesh shape — quality check**

The long axis (LA) was defined as the line connecting the centre of the mitral valve and the left ventricular apex. The short axis (SA) was then computed as the perpendicular axis at the midpoint of the long axis with the largest length spanning the left ventricle. Afterwards, the sphericity index (SI) of the left ventricle was computed using **SI = SA/LA**. For the meshes with a flattening distortion, the SI was zero or very close to zero. For accuracy and robust exclusion, the meshes with SI greater than ±3 SD from the population mean were omitted. This was ~0.5% of the total number of surface meshes. This is shown below in Figure A.


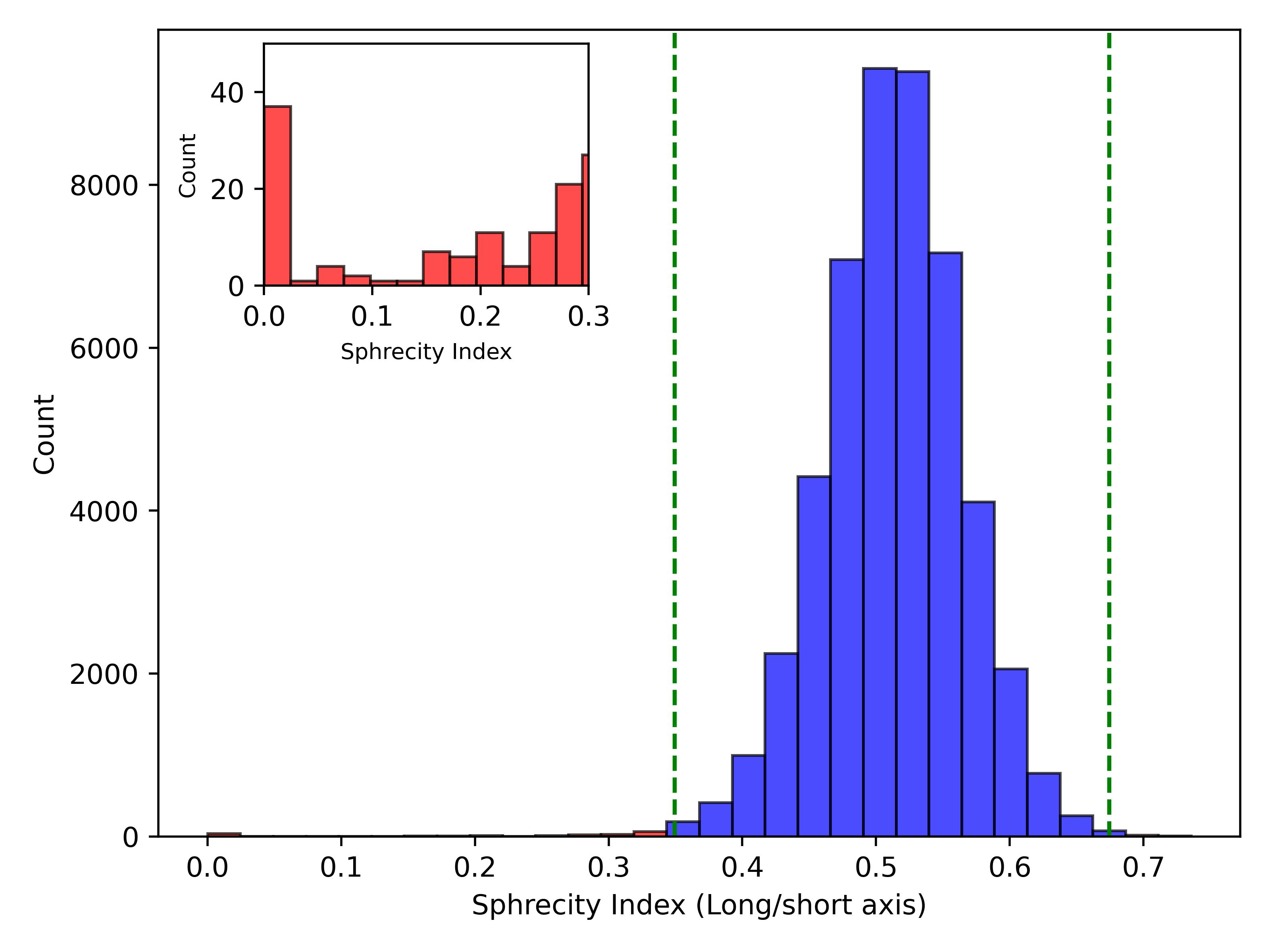

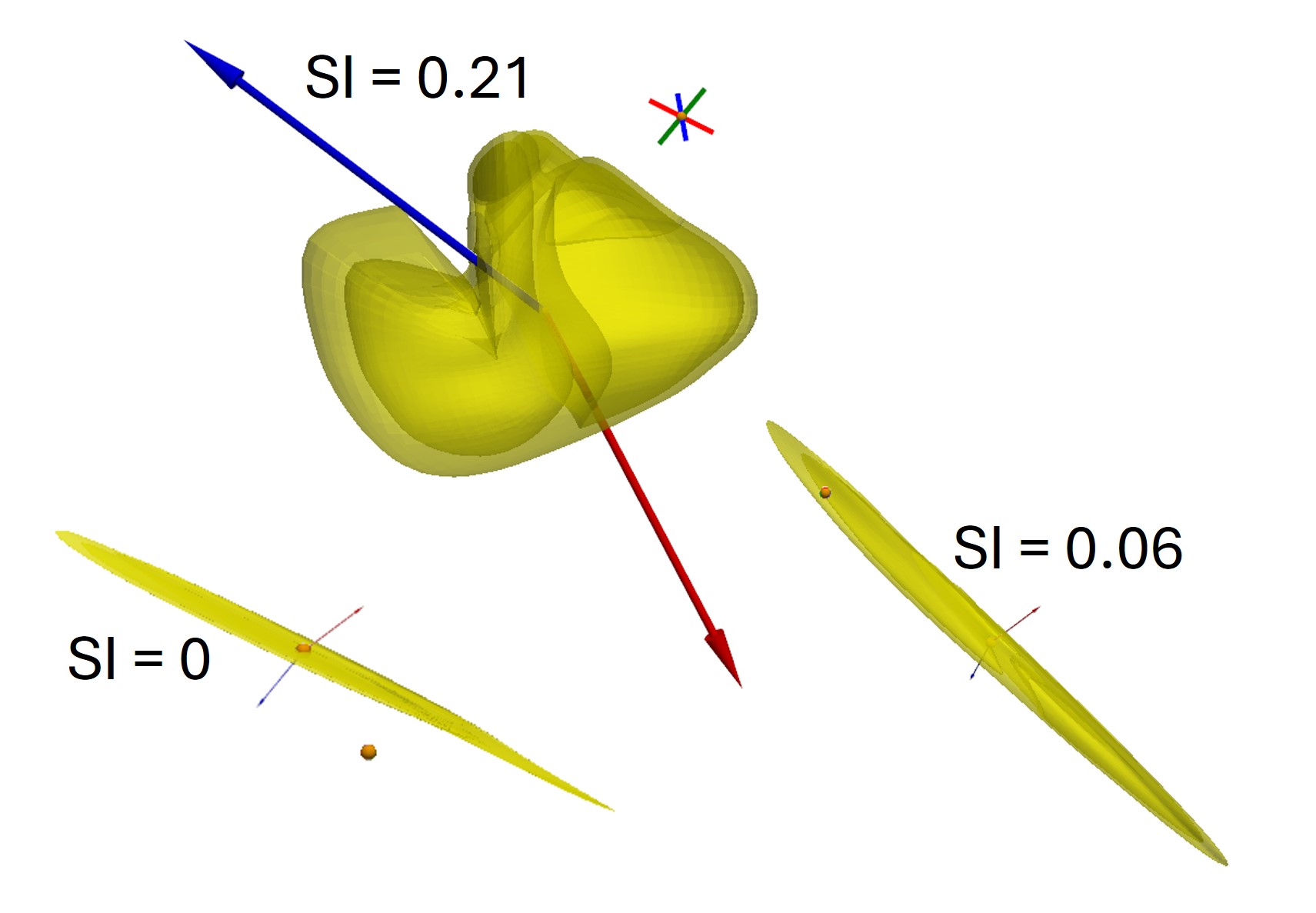


Fig A. LHS: Histogram of sphericity index (SI) distributions. Dashed green lines shows 3 SD thresholds and the inset shows the meshes with SI > 3 SD away from the mean. RHS: 3 examples of abnormal meshes that were successfully excluded via SI exclusion criteria.

**S1.2. UK BioBank — Diagnostic-based patient filtering**

UK Biobank Fields 41270 and 41280, which are summaries derived from the linked Hospital Episode Statistics (HES) Inpatient Records, were used in this study. These include historical records and are updated on a regular basis. Field 41270 provides diagnoses recorded across all hospital inpatient records for each participant, while Field 41280 contains the corresponding dates of these diagnoses. We aligned each diagnosis code with the date at which it was made. We used field 53 for the date of attendance at the assessment centre. Instance 2 was used as this was the imaging visit when the cMRI and ECG were acquired. Only diagnoses made within one year after the assessment were included in the analysis. Subjects with diagnoses of ’Diseases of the Circulatory system’ (I00-I99) were excluded.

**S1.3 Axes alignment & Coordinate systems**

The biventricular mesh reconstruction aligns with the DICOM coordinate system. An alignment (i.e. a purely rotational transformation) exists between the DICOM and VCG coordinate systems. A RAI (Right to left, Anterior to posterior, Inferior to superior) convention for the XYZ axes is adopted to present results, a convention that will not affect the results, either in terms of trends or coupling. Conventions on axes were also established: the anatomical axis extends from the apex (bottom of the biventricular anatomy) to the base (top), whereas the electrical axis aligns with the direction of depolarisation. As a result, the electrical axis points towards the left (+x) and apical (−z) directions—denoting LV depolarisation and shows the most variations in the transverse and sagittal planes (see bottom panels in Figure B LHS). The anatomical axis generally points towards the right (+x) and basal (+z) directions.


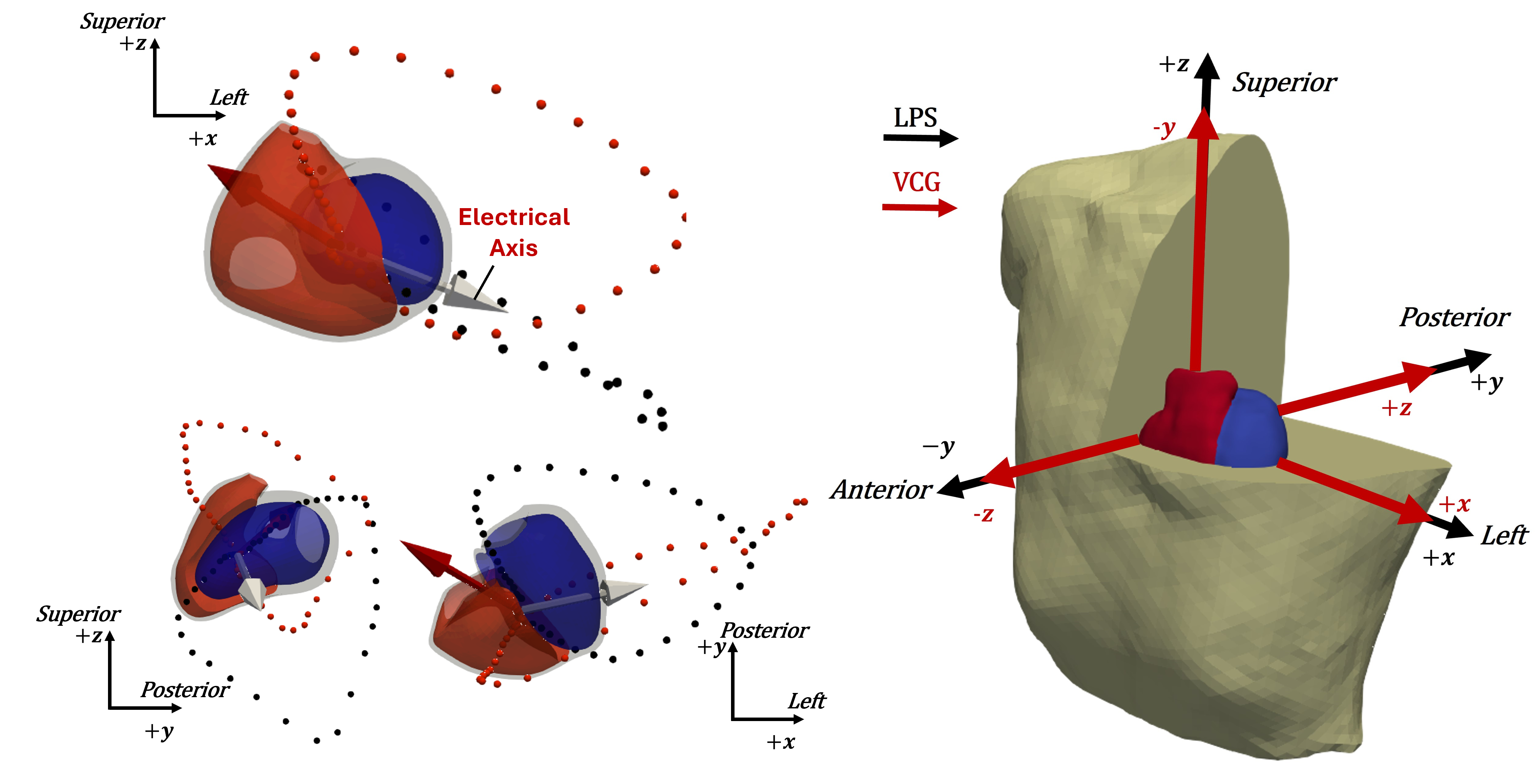


Fig B. LHS: Biventricular anatomy and VCG in the 3 anatomical planes with VCG (red) and DICOM (black). Electrical and anatomical axes are also shown as arrows. RHS: Torso and biventricular geometry with a visualisation of the 2 coordinate systems.

**S1.4 MLR further results**

Detailed results of the pairwise correlations and variance inflation factors (VIF) of predictor variables for both MLRs performed are shown below in Tables 1,2 and 4, showing no significant multicollinearity. Goodness-of-fit indicators are in Tables 3 and 5.

*MLR 1 – Demographics and orientation metrics*

Details on the results of the MLR concerning the relationship between each orientation metric and the 3 demographic variables are shown below.

| Feature | VIF |
| --- | --- |
| BMI | 1.017568 |
| Age | 1.008629 |
| Sex | 1.018822 |

Table A. Pairwise Pearson r correlation values. Table B. VIF values for the 3 predictor values, all being <5.

|  | BMI | Age | Sex |
| --- | --- | --- | --- |
| BMI | 1.00 | -0.06 | 0.11 |
| Age | -0.06 | 1.00 | 0.07 |
| Sex | 0.11 | 0.07 | 1.00 |

Table C. Goodness-of-fit indicators for each model where the predictor variables were BMI, Age and Sex.

| Model | R2 | RMSE |
| --- | --- | --- |
| theta_A | 0.295 | 7.382 |
| theta_E | 0.148 | 13.029 |
| phi_A | 0.165 | 6.698 |
| phi_E | 0.027 | 17.926 |
| AE_angle | 0.015 | 16.619 |

*MLR 2 – Anatomical orientation and effect variables*

Details on the results of the MLR concerning the relationship between the frontal orientation metric and predictor variables are shown below.

Table D. Multicollinearity testing results. Pairwise correlations and VIF for all predictor variables.

| Healthy | | | | | | | Healthy | |
| --- | --- | --- | --- | --- | --- | --- | --- | --- |
|  | BMI | Sex | Age | BSA | Arterial stiffness | MAP | Feature | VIF |
| BMI | 1.000 | 0.104 | -0.063 | 0.608 | 0.061 | 0.155 | BMI | 2.065 |
| Sex | 0.104 | 1.000 | 0.065 | 0.638 | 0.106 | 0.060 | Sex | 2.227 |
| Age | -0.063 | 0.065 | 1.000 | -0.099 | 0.036 | 0.168 | Age | 1.079 |
| BSA | 0.608 | 0.638 | -0.099 | 1.000 | 0.110 | 0.094 | BSA | 3.490 |
| Arterial stiffness | 0.061 | 0.106 | 0.036 | 0.110 | 1.000 | 0.069 | Arterial stiffness | 1.019 |
| MAP | 0.155 | 0.060 | 0.168 | 0.094 | 0.069 | 1.000 | MAP | 1.063 |
| Primary HT | | | | | | | Primary HT | |
|  | BMI | Sex | Age | BSA | Arterial stiffness | MAP | Feature | VIF |
| BMI | 1.000 | -0.015 | -0.188 | 0.579 | 0.020 | 0.030 | BMI | 2.072 |
| Sex | -0.015 | 1.000 | 0.047 | 0.577 | 0.062 | -0.046 | Sex | 2.139 |
| Age | -0.188 | 0.047 | 1.000 | -0.202 | 0.013 | 0.065 | Age | 1.095 |
| BSA | 0.579 | 0.577 | -0.202 | 1.000 | 0.058 | -0.021 | BSA | 3.249 |
| Arterial stiffness | 0.020 | 0.062 | 0.013 | 0.058 | 1.000 | 0.048 | Arterial stiffness | 1.007 |
| MAP | 0.030 | -0.046 | 0.065 | -0.021 | 0.048 | 1.000 | MAP | 1.011 |

Table E. Goodness-of-fit indicators for the model predicting frontal orientation in healthy vs hypertensive group. Note that R2 of hypertensive group was 0.18 in out-of-sample performance (test/train)

| Group | R2 | RMSE |
| --- | --- | --- |
| Healthy | 0.3 | 7.382 |
| Hypertensive | 0.11 | 10.16 |

**S1.5. Further results**


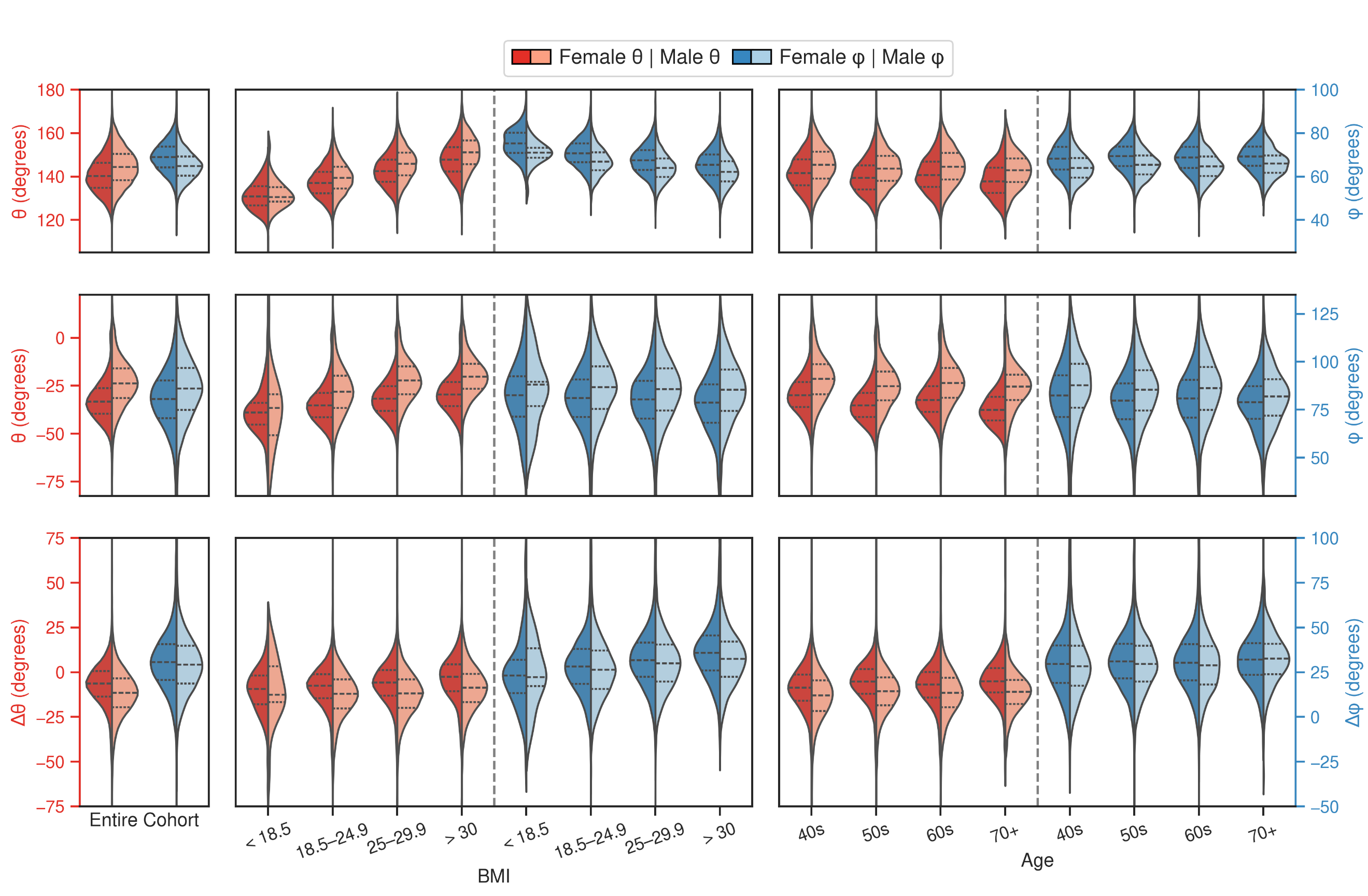


Fig C. Violin plot representation of $\theta_{Anatomical}$, $\theta_{Electrical}$, $\phi_{Anatomical}$, $\phi_{Electrical}$, $\Delta\theta$ and $\Delta\phi$, divided by BMI and age groups along with their respective sex distributions. According to pairwise comparisons, all BMI groups are significantly different from each other for $\Delta\phi$ and not $\Delta\theta$.

Table F. Pearson correlation coefficients for the angular measures of the anatomical and electrical axes. Sex is reported as 0 for Female and 1 for male. *All correlations are statistically significant at p < 0.001.

|  | Anatomical | | Electrical | |
| --- | --- | --- | --- | --- |
|  | $\theta$ | $\phi$ | $\theta$ | $\phi$ |
| BMI | 0.50 | -0.31 | 0.22 | -0.02 |
| Sex | 0.21 | -0.28 | 0.29 | 0.15 |
| Age | 0.12 | -0.10 | 0.16 | 0.04 |


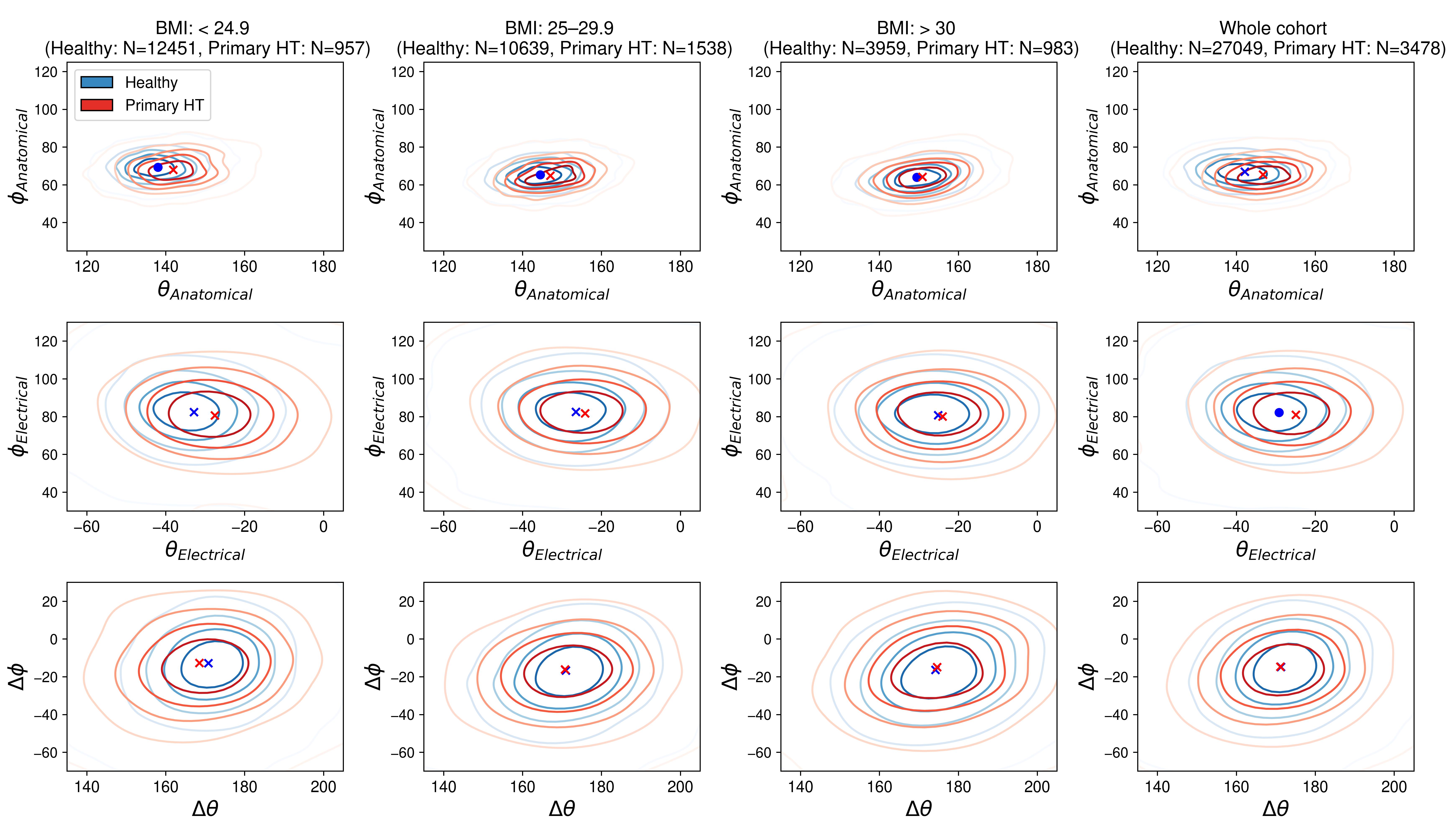


Fig D. Kernel density estimates of the respective distributions of healthy and disease cohorts, broken down by BMI group. “x” markers represent the median of each group.


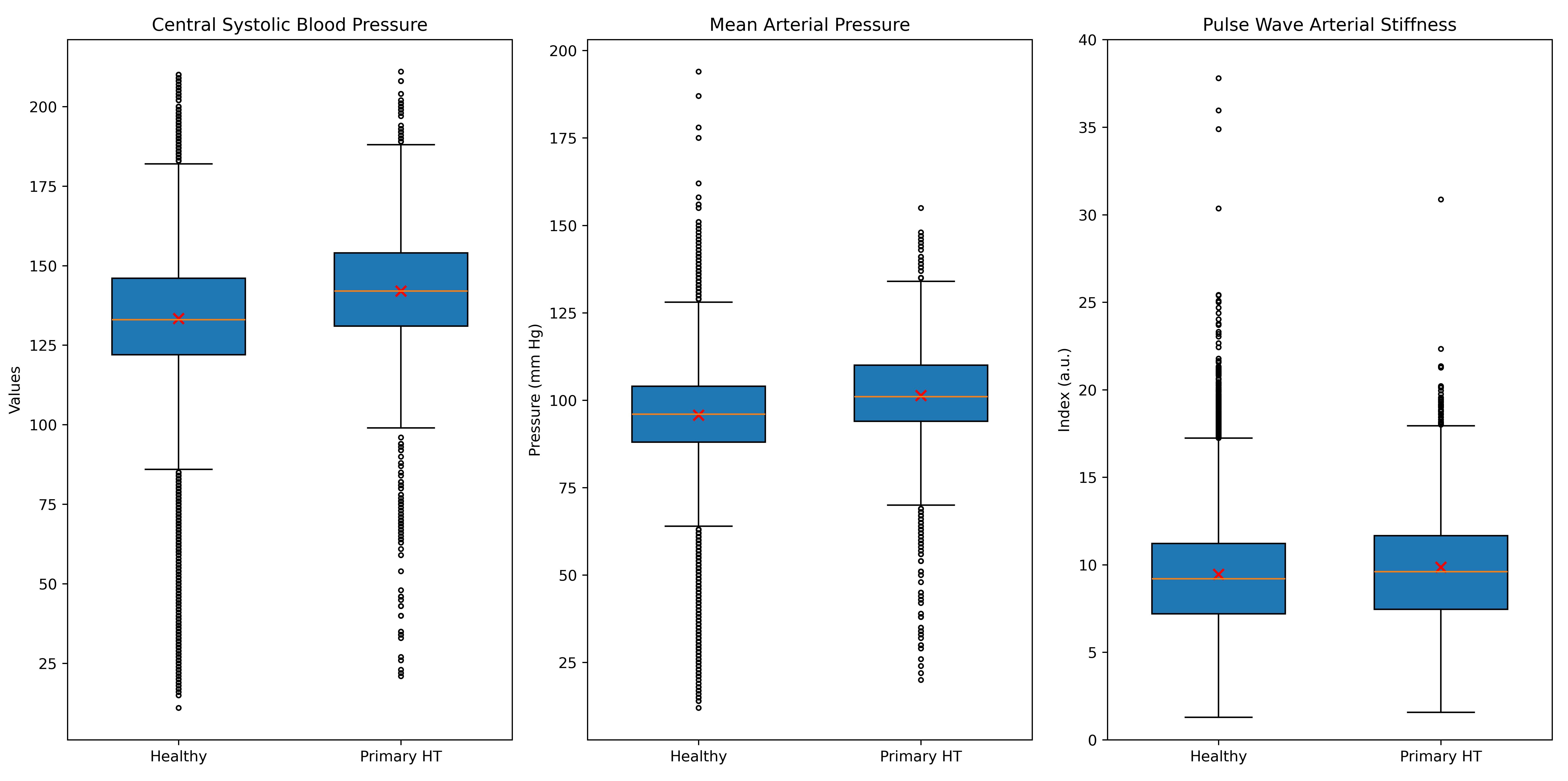


Fig E. Comparison of CSBP, MAP, and AS between the healthy and disease (primary HT) cohorts. All metrics are significantly different between the 2 cohorts at p < 0.001, where the disease cohort had higher means.

Table G. The most significant correlations based on -log p-value between the axes metrics and the UK Biobank phenotypes. (Only those at MRI visit (instance 2) are shown.

| Rank | UKBB Field | Related Phenotype | Log p-value | Correlation |
| --- | --- | --- | --- | --- |
| *θ*_Anatomical | | | | |
| 1 | 22336-2.0 | R axis | 127.793 | -0.170 |
| 2 | 22337-2.0 | T axis | 107.962 | -0.145 |
| 3 | 22407-2.0 | Visceral adipose tissue volume (VAT) | 75.681 | 0.114 |
| 4 | 22335-2.0 | P axis | 67.358 | -0.115 |
| 5 | 4079-2.1 | Diastolic blood pressure, automated reading | 42.868 | 0.093 |
| 6 | 4079-2.0 | Diastolic blood pressure, automated reading | 39.750 | 0.088 |
| 7 | 24120-2.0 | Ascending aorta distensibility | 28.469 | -0.084 |
| 8 | 54-2.0 | UK Biobank assessment centre | 24.955 | 0.064 |
| 9 | 3062-2.2 | Forced vital capacity (FVC) | 21.706 | -0.078 |
| 10 | 3062-2.0 | Forced vital capacity (FVC) | 20.004 | -0.064 |
| 11 | 22410-2.0 | Total trunk fat | 19.508 | 0.065 |
| 12 | 3062-2.1 | Forced vital capacity (FVC) | 18.727 | -0.062 |
| 13 | 12683-2.0 | End systolic pressure during PWA | 17.703 | 0.056 |
| 14 | 12687-2.0 | Mean arterial pressure during PWA | 15.479 | 0.052 |
| 15 | 12683-2.1 | End systolic pressure during PWA | 14.786 | 0.052 |
| 16 | 22330-2.0 | PQ interval | 14.442 | 0.051 |
| 17 | 12684-2.0 | End systolic pressure index during PWA | 14.129 | 0.051 |
| 18 | 12698-2.0 | Diastolic brachial blood pressure | 14.072 | 0.049 |
| 19 | 12675-2.0 | Diastolic brachial blood pressure during PWA | 13.852 | 0.049 |
| 20 | 12687-2.1 | Mean arterial pressure during PWA | 13.754 | 0.050 |
| φ_Anatomical | | | | |
| 1 | 24100-2.0 | LV end diastolic volume | 78.768 | -0.131 |
| 2 | 22333-2.0 | RR interval | 68.301 | -0.112 |
| 3 | 22426-2.0 | Average heart rate | 67.310 | 0.121 |
| 4 | 12336-2.0 | Ventricular rate | 62.987 | 0.103 |
| 5 | 22331-2.0 | QT interval | 60.713 | -0.105 |
| 6 | 22423-2.0 | LV stroke volume | 58.884 | -0.113 |
| 7 | 12679-2.0 | Number of beats in waveform average for PWA | 57.958 | 0.103 |
| 8 | 24101-2.0 | LV end systolic volume | 57.774 | -0.112 |
| 9 | 22334-2.0 | PP interval | 56.612 | -0.101 |
| 10 | 22338-2.0 | QRS num | 53.084 | 0.098 |
| 11 | 12673-2.1 | Heart rate during PWA | 50.688 | 0.097 |
| 12 | 12679-2.1 | Number of beats in waveform average for PWA | 48.966 | 0.095 |
| 13 | 12673-2.0 | Heart rate during PWA | 48.638 | 0.094 |
| 14 | 24105-2.0 | 24105-2.0 | 46.502 | -0.100 |
| 15 | 102-2.1 | Pulse rate, automated reading | 44.803 | 0.095 |
| 16 | 4194-2.0 | Pulse rate | 38.026 | 0.084 |
| 17 | 102-2.0 | Pulse rate, automated reading | 37.495 | 0.086 |
| 18 | 12340-2.0 | QRS duration | 32.299 | -0.073 |
| 19 | 20015-2.0 | Sitting height | 27.592 | -0.067 |
| 20 | 23127-2.0 | Trunk fat percentage | 25.275 | 0.065 |
| *θ*_Electrical | | | | |
| 1 | 12340-2.0 | QRS duration | 76.364 | 0.113 |
| 2 | 22336-2.0 | R axis | 46.576 | -0.102 |
| 3 | 22420-2.0 | LV ejection fraction | 13.208 | -0.052 |
| 4 | 22330-2.0 | PQ interval | 12.816 | 0.048 |
| 5 | 22331-2.0 | QT interval | 7.947 | 0.036 |
| 6 | 22332-2.0 | QTC interval | 7.533 | 0.035 |
| 7 | 24103-2.0 | LV ejection fraction | 7.345 | -0.038 |
| 8 | 4079-2.0 | Diastolic blood pressure, automated reading | 6.699 | 0.035 |
| 9 | 24101-2.0 | LV end systolic volume | 6.601 | 0.036 |
| 10 | 4079-2.1 | Diastolic blood pressure, automated reading | 4.639 | 0.028 |
| φ_Electrical | | | | |
| 1 | 22337-2.0 | T axis | 61.013 | -0.108 |
| 2 | 22330-2.0 | PQ interval | 24.001 | -0.067 |
| 3 | 22336-2.0 | R axis | 17.470 | -0.062 |
| 4 | 24101-2.0 | LV end systolic volume | 16.902 | -0.060 |
| 5 | 24100-2.0 | LV end diastolic volume | 16.787 | -0.059 |
| 6 | 12340-2.0 | QRS duration | 16.089 | -0.051 |
| 7 | 22333-2.0 | RR interval | 14.415 | -0.050 |
| 8 | 22334-2.0 | PP interval | 12.839 | -0.047 |
| 9 | 4080-2.1 | Systolic blood pressure, automated reading | 11.587 | -0.047 |
| 10 | 4080-2.0 | Systolic blood pressure, automated reading | 11.223 | -0.046 |
| 11 | 12336-2.0 | Ventricular rate | 11.161 | 0.042 |
| 12 | 12683-2.1 | End systolic pressure during PWA | 11.089 | -0.045 |
| 13 | 24105-2.0 | 24105-2.0 | 10.855 | -0.047 |
| 14 | 24120-2.0 | Ascending aorta distensibility | 10.241 | 0.049 |
| 15 | 22338-2.0 | QRS num | 9.857 | 0.041 |
| 16 | 12683-2.0 | End systolic pressure during PWA | 9.003 | -0.039 |
| 17 | 12338-2.0 | P duration | 8.448 | 0.037 |
| 18 | 12673-2.0 | Heart rate during PWA | 8.275 | 0.037 |
| 19 | 54-2.0 | UK Biobank assessment centre | 8.177 | -0.035 |
| 20 | 22421-2.0 | LV end diastolic volume | 8.123 | -0.040 |
| Δ*θ* | | | | |
| 1 | 22336-2.0 | R axis | 26.069 | 0.076 |
| 2 | 12340-2.0 | QRS duration | 17.931 | -0.054 |
| 3 | 22331-2.0 | QT interval | 9.866 | -0.041 |
| 4 | 24101-2.0 | LV end systolic volume | 7.244 | -0.038 |
| 5 | 22420-2.0 | LV ejection fraction | 6.583 | 0.036 |
| 6 | 24103-2.0 | LV ejection fraction | 6.100 | 0.034 |
| 7 | 22333-2.0 | RR interval | 5.187 | -0.029 |
| 8 | 102-2.0 | Pulse rate, automated reading | 5.012 | 0.029 |
| 9 | 12673-2.0 | Heart rate during PWA | 4.911 | 0.028 |
| 10 | 4194-2.0 | Pulse rate | 4.647 | 0.028 |
| Δφ | | | | |
| 1 | 22337-2.0 | T axis | 88.593 | 0.131 |
| 2 | 22330-2.0 | PQ interval | 15.922 | 0.054 |
| 3 | 22336-2.0 | R axis | 15.396 | 0.058 |
| 4 | 12338-2.0 | P duration | 14.093 | -0.048 |
| 5 | 54-2.0 | UK Biobank assessment centre | 13.415 | 0.046 |
| 6 | 4080-2.1 | Systolic blood pressure, automated reading | 8.760 | 0.040 |
| 7 | 4080-2.0 | Systolic blood pressure, automated reading | 8.610 | 0.040 |
| 8 | 12683-2.1 | End systolic pressure during PWA | 8.575 | 0.039 |
| 9 | 22420-2.0 | LV ejection fraction | 8.168 | -0.040 |
| 10 | 4079-2.0 | Diastolic blood pressure, automated reading | 7.918 | 0.038 |
| 11 | 4079-2.1 | Diastolic blood pressure, automated reading | 7.178 | 0.036 |
| 12 | 12683-2.0 | End systolic pressure during PWA | 6.569 | 0.033 |
| 13 | 12677-2.0 | Central systolic blood pressure during PWA | 5.304 | 0.029 |
| 14 | 24120-2.0 | Ascending aorta distensibility | 5.273 | -0.034 |
| 15 | 12687-2.0 | Mean arterial pressure during PWA | 5.268 | 0.029 |
| 16 | 22335-2.0 | P axis | 5.141 | -0.030 |
| 17 | 12697-2.0 | Systolic brachial blood pressure | 4.904 | 0.028 |
| 18 | 12340-2.0 | QRS duration | 4.886 | 0.027 |

Table H. The most significant correlations based on -log p-value between the axes metrics and the prevalent + incident clinical diagnoses.

| Rank | Phecode | Phecode Category | Phecode Group | Log p-value | Correlation |
| --- | --- | --- | --- | --- | --- |
|  | *θ*_Anatomical | | | | |
| 1 | 401.1 | circulatory system | Essential hypertension | 28.34 | 0.06 |
| 2 | 272.11 | endocrine/metabolic | Hypercholesterolemia | 5.88 | 0.02 |
| 3 | 411.4 | circulatory system | Coronary atherosclerosis | 4.89 | 0.02 |
| 4 | 411.8 | circulatory system | Other chronic ischemic heart disease, unspecified | 4.62 | 0.02 |
|  | θ_Electrical | | | | |
| 1 | 278.1 | endocrine/metabolic | Obesity | 8.44 | -0.03 |
| 2 | 600 | genitourinary | Hyperplasia of prostate | 7.65 | -0.03 |
| 3 | 250.2 | endocrine/metabolic | Type 2 diabetes | 6.64 | -0.03 |
| 4 | 426.31 | circulatory system | Right bundle branch block | 6.42 | 0.03 |
| 5 | 306 | mental disorders | Other mental disorder | 6.39 | -0.03 |
| 6 | 411.4 | circulatory system | Coronary atherosclerosis | 6.08 | -0.03 |
| 7 | 272.11 | endocrine/metabolic | Hypercholesterolemia | 5.35 | -0.02 |
| 8 | 411.3 | circulatory system | Angina pectoris | 5.21 | -0.02 |
| 9 | 411.8 | circulatory system | Other chronic ischemic heart disease, unspecified | 4.91 | -0.02 |
|  | φ_Anatomical | | | | |
| 1 | 427.2 | circulatory system | Atrial fibrillation and flutter | 21.02 | -0.05 |
| 2 | 550.2 | digestive | Diaphragmatic hernia | 12.19 | 0.04 |
| 3 | 416 | circulatory system | Cardiomegaly | 10.08 | -0.03 |
| 4 | 395.1 | circulatory system | Nonrheumatic mitral valve disorders | 8.35 | -0.03 |
| 5 | 394.2 | circulatory system | Mitral valve disease | 8.15 | -0.03 |
| 6 | 428.1 | circulatory system | Congestive heart failure (CHF) NOS | 7.33 | -0.03 |
| 7 | 428.2 | circulatory system | Heart failure NOS | 5.70 | -0.02 |
| 8 | 411.8 | circulatory system | Other chronic ischemic heart disease, unspecified | 5.40 | -0.02 |
| 9 | 414 | circulatory system | Other forms of chronic heart disease | 5.21 | -0.02 |
| 10 | 530.14 | digestive | Reflux esophagitis | 5.16 | 0.02 |
|  | φ_Electrical | | | | |
| 1 | 426.32 | circulatory system | Left bundle branch block | 19.28 | -0.05 |
| 2 | 401.1 | circulatory system | Essential hypertension | 15.54 | -0.04 |
| 3 | 426.31 | circulatory system | Right bundle branch block | 13.54 | 0.04 |
| 4 | 411.2 | circulatory system | Myocardial infarction | 10.80 | -0.03 |
| 5 | 428.2 | circulatory system | Heart failure NOS | 10.72 | -0.03 |
| 6 | 414 | circulatory system | Other forms of chronic heart disease | 6.98 | -0.03 |
| 7 | 427.2 | circulatory system | Atrial fibrillation and flutter | 6.53 | -0.03 |
| 8 | 426.21 | circulatory system | First degree AV block | 6.23 | -0.03 |
| 9 | 411.4 | circulatory system | Coronary atherosclerosis | 6.22 | -0.03 |
| 10 | 411.8 | circulatory system | Other chronic ischemic heart disease, unspecified | 5.75 | -0.02 |
| 11 | 416 | circulatory system | Cardiomegaly | 4.88 | -0.02 |
| 12 | 395.1 | circulatory system | Nonrheumatic mitral valve disorders | 4.81 | -0.02 |
| 13 | 394.2 | circulatory system | Mitral valve disease | 4.70 | -0.02 |
|  | Δφ | | | | |
| 1 | 426.31 | circulatory system | Right bundle branch block | 19.85 | -0.05 |
| 2 | 426.32 | circulatory system | Left bundle branch block | 18.63 | 0.05 |
| 3 | 401.1 | circulatory system | Essential hypertension | 9.40 | 0.03 |
| 4 | 428.2 | circulatory system | Heart failure NOS | 5.79 | 0.02 |
| 5 | 411.2 | circulatory system | Myocardial infarction | 5.66 | 0.02 |
| 6 | 426.21 | circulatory system | First degree AV block | 4.61 | 0.02 |
